# Genetic evidence informs the direction of therapeutic modulation in drug development

**DOI:** 10.1038/s44386-025-00027-0

**Published:** 2025-10-01

**Authors:** Robert Chen, Áine Duffy, Joshua K. Park, David Stein, Avner Schlessinger, Yuval Itan, Benjamin S. Glicksberg, Rory J. Tinker, Daniel M. Jordan, Ghislain Rocheleau, Ron Do

**Affiliations:** 1 The Charles Bronfman Institute for Personalized Medicine, Icahn School of Medicine at Mount Sinai, New York, NY USA; 2 Department of Genetics and Genomic Sciences, Icahn School of Medicine at Mount Sinai, New York, NY USA; 3 Medical Scientist Training Program, Icahn School of Medicine at Mount Sinai, New York, NY USA; 4 Windreich Department of Artificial Intelligence and Human Health, Icahn School of Medicine at Mount Sinai, New York, NY USA; 5 Center for Genomic Data Analytics, Icahn School of Medicine at Mount Sinai, New York, NY USA; 6 Department of Pharmacological Sciences, Icahn School of Medicine at Mount Sinai, New York, NY USA; 7 Mindich Child Health and Development Institute, Icahn School of Medicine at Mount Sinai, New York, NY USA; 8 Department of Pediatrics, Division of Medical Genetics and Genomic Medicine, Vanderbilt University Medical Center, Nashville, TN USA

**Keywords:** Target identification, Target validation

## Abstract

Determining the correct direction of effect (DOE), whether to increase or decrease the activity of a drug target, is essential for therapeutic success. We introduce a framework to predict DOE at gene and gene-disease levels using gene and protein embeddings and genetic associations across the allele frequency spectrum, respectively. Specifically, we predict: (1) DOE-specific druggability for 19,450 protein-coding genes with a macro-averaged area under the receiver operating characteristic curve (AUROC) of 0.95; (2) isolated DOE among 2553 druggable genes with a macro-averaged AUROC of 0.85; and (3) gene-disease-specific DOE for 47,822 gene-disease pairs with a macro-averaged AUROC of 0.59, with performance improving with genetic evidence availability. Our predictions outperform existing approaches, are associated with clinical trial success, and identify novel therapeutic opportunities. We uncover genetic and functional differences between activator and inhibitor targets, allowing DOE inference independent of disease context. This framework represents a valuable tool for target selection and drug development.

## Introduction

Successful target-based drug development requires establishing the target’s causality in disease, its druggability, potential safety issues, and the appropriate direction of effect (DOE; whether to activate or inhibit the target)^1,2^. Prevalent issues with these criteria may explain the 90% failure rate of clinical drug development^3^. Human genetic evidence supporting gene-disease causality has been associated with a 2.6-fold increase in drug development success^4^, and existing scores successfully use multiple lines of evidence to prioritize targets^5–8^. Druggability represents the ability to modulate a target to elicit a therapeutic effect, and machine learning models can accurately predict druggability using gene-level features^9^. Genetic features like tissue specificity, genetic associations, and constraint also predict target-specific adverse effects, improving therapeutic safety^10,11^. Although determining the correct DOE for target modulation is equally important, as incorrect DOE determination leads to suboptimal therapeutic strategies and adverse effects^12^, approaches to predict DOE are lacking.

Most existing DOE prediction approaches focus on determining the mechanism of action of specific drug candidates using perturbation data^13–15^, rather than predicting the correct DOE prior to compound development. Existing models like DrugnomeAI predict gene-level druggability but do not differentiate between activators and inhibitors^9^. However, because prior studies accurately predicted DOE-adjacent traits like dosage sensitivity^16^, mode of inheritance^17^, and gain-of-function (GOF) versus loss-of-function (LOF) disease mechanisms^18,19^, we hypothesize it is possible to predict the suitability of a gene for modulation by activator and inhibitor drugs.

Simultaneously, human genetics informs DOE by demonstrating how GOF and LOF mutations or gene expression changes affect disease risk through dose-response relationships^1^. These patterns guide drug development by identifying modulation patterns that mimic protective genetic effects. For example, GOF mutations increasing disease risk suggest inhibitor drugs are necessary. However, efforts to predict gene-disease-specific DOE also remain limited. Our prior genetic priority score (GPS) framework incorporated effect directions from genetic variants across the allele frequency spectrum (common, rare, ultrarare) to predict both drug indications and DOE^6^, but its reliance on UK Biobank data limited its accuracy and generalizability. Recently, Open Targets integrated DOE predictions from eight data sources, covering over 2.3 million assessments for 865,816 target-disease pairs^8^. While this represents a significant advancement, the accuracy and utility of these predictions remain unvalidated.

We address these gaps by developing three new genetics-informed DOE prediction models (Fig. S1). First, we predict DOE-specific druggability for 19,450 protein-coding genes. These predictions aim to expand the druggable genome in a DOE-specific manner and reduce the imbalance between activator versus inhibitor targets, with therapeutic activation being more challenging to achieve than inhibition^20^. Second, we predict DOE independent of druggability for 4732 known and predicted druggable genes. These predictions represent whether it is therapeutically useful to modulate a target in a certain direction across all diseases. In contrast to the first two disease-agnostic gene-level models, the third model predicts gene-disease-specific DOE among 47,822 gene-disease pairs using human genetics features.

All three models incorporate methodological advances. For gene-level models, beyond standard tabular features like constraint and essentiality, we include GenePT embeddings of NCBI gene summaries and ProtT5 embeddings of amino acid sequences^21,22^. These continuous representations of gene and protein function improve model performance. For the gene-disease-specific model, we incorporate genetic associations across the allele frequency spectrum from up to five datasets. This represents an allelic series, where different variants within the same gene exert graded effects on disease risk, modeling a dose-response relationship that informs DOE^1^. While allelic series have traditionally supported drug indications, our approach uses them to generate probabilistic DOE predictions. Together, these three models provide a comprehensive framework for DOE prediction, offering insights to inform target selection and accelerate drug development.

## Results

### Characteristics of drugs and druggable genes

**Fig. 1:**
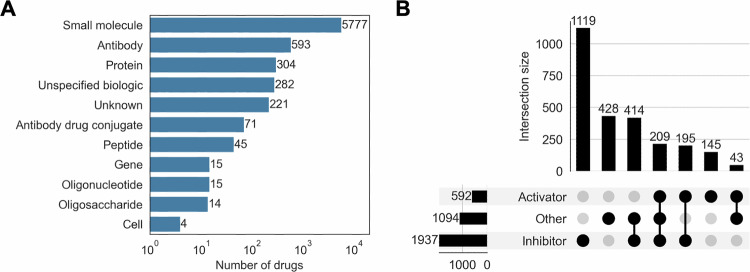
Characteristics of drugs and targets. **A** Number of drugs by type. The x-axis is in log_10_ scale. **B** Number of genes targeted by activator drugs, inhibitor drugs, and/or drugs with other mechanisms. One gene can be targeted by multiple drugs with different DOEs. A total of 2553 genes are targeted by at least one drug in our dataset.

We identified up to 7341 unique drugs with specified mechanisms of action from five sources. Of these, 46% were in phase IV (approved), 29% were in phase I to III clinical trials, and 25% were under an unspecified phase of investigation. The most common drug types were small molecules (78.7%) and antibodies (8.1%) (Fig. 1A), and 54.7% of drugs targeted only a single gene (Fig. S2A). A total of 2553 protein-coding genes were targeted by at least one of the 7341 drugs. Of these genes, 1937 (75.9%) were targeted by inhibitor drugs, 592 (23.2%) by activator drugs, 1094 (42.9%) by drugs with other mechanisms, and 404 (15.8%) by both activator and inhibitor drugs (Fig. 1B). Examples of mechanisms in the other category included binding agents, mixed agonist/antagonists, and gene/protein replacement therapies (Fig. S2B).

### Activator and inhibitor drug targets have distinct characteristics

**Fig. 2:**
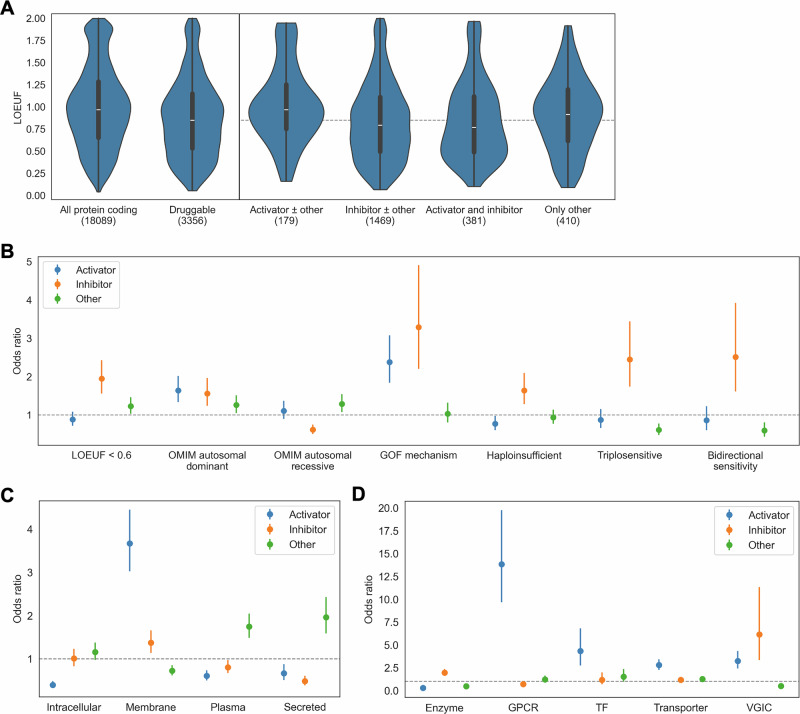
Characteristics of activator and inhibitor drug targets. **A** Violin plot of loss-of-function observed/expected upper bound fraction (LOEUF) scores for different gene categories. Each gene can be targeted by drugs with different DOEs; for example, “Activator ± other” indicates genes targeted by activator drugs, some of which may also be targeted by drugs with other mechanisms. The dashed line indicates the median LOEUF for druggable genes. Numbers in parentheses indicate the number of genes in each category with non-missing values. **B** Odds ratios for enrichment of activator, inhibitor, and other drug mechanisms across different gene categories. **C** Odds ratios for enrichment of activator, inhibitor, and other drug mechanisms across protein localization categories. **D** Odds ratios for enrichment of activator, inhibitor, and other drug mechanisms across protein functional categories, including enzymes, G protein-coupled receptors (GPCRs), transcription factors (TFs), transporters, and voltage-gated ion channels (VGICs). For **B**–**D**, we calculated odds ratios using logistic regression among 2553 druggable genes with known drug DOEs. We performed separate regressions for each gene or protein category (independent variables) and for each DOE (dependent variables). It is possible for all three odds ratios to be positive (e.g., OMIM autosomal dominant) because one gene can be targeted by drugs with different DOEs. Error bars represent 95% confidence intervals.

A prior study of 383 approved drug targets showed that drug targets are more constrained compared to all genes, with inhibitor drug targets being more constrained than activator drug targets^23^. We replicated these findings in our larger dataset including investigational drugs, finding that drug targets had significantly lower LOF observed/expected upper bound fraction (LOEUF) scores compared to all protein-coding genes (p_rank-sum_ = 9.4 × 10^−44^) and that inhibitor targets had lower LOEUF scores compared to activator targets (p_rank-sum_ = 8.5 × 10^−8^) (Fig. 2A). LOEUF quantifies a gene’s intolerance to LOF variants, with lower scores indicating stronger selective constraint against inactivation^24^. Drug targets also had higher predicted dosage sensitivity [i.e., increased susceptibility to phenotypic consequences from reduced (haploinsufficiency) or increased (triplosensitivity) gene dosage] compared to all protein-coding genes^16^, with inhibitor targets having higher predictions than activator targets (Fig. S3A, B). Nevertheless, the wide distributions of constraint and dosage sensitivity metrics for each target category suggest that neither low nor high metrics preclude druggability or a specific DOE.

While it is counterintuitive that inhibitor targets are more LOF intolerant, since inhibitor drugs achieve efficacy by mimicking LOF, this is likely due to confounding factors^23^. For example, chemotherapies inhibit essential genes, and constrained compared to unconstrained inhibitor targets (LOEUF cutoff = 0.6) were enriched for DepMap common essential genes [odds ratio (OR) = 4.3, 95% confidence interval (CI) 3.2–5.8]. Inhibitors can also treat GOF or overexpression-related phenotypes associated with these targets, and indeed, constrained inhibitor targets were also enriched for GoFCards GOF disease mechanisms (OR = 2.2, 95% CI = 1.7–2.9) and predicted triplosensitivity (OR = 10.8, 95% CI 8.0–14.6).

Besides constraint and dosage sensitivity, genes involved in autosomal dominant disorders were enriched for both activator and inhibitor mechanisms, whereas genes involved in autosomal recessive disorders were depleted only of inhibitor mechanisms (Fig. 2B). This is likely because many autosomal recessive disorders have LOF mechanisms, whereas autosomal dominant disorders have more diverse mechanisms^25^. In parallel, genes causing disease via GOF mechanisms were more enriched for inhibitors compared to activator mechanisms (Fig. 2B). Protein localization and class also predicted DOE, consistent with known patterns (Fig. 2C, D); for example, G protein-coupled receptors were enriched for activators. Overall, despite the complexity of multiple influencing factors, the significant associations of DOE with gene-level characteristics suggest that DOE itself can be accurately predicted at the gene level.

### Predicting overall and DOE-specific druggability

We trained gene-level models using 41 tabular features (Supplementary Data 1), 256-dimensional gene embeddings, and 128-dimensional protein embeddings. To assess the utility of embeddings for drug development tasks, we first predicted the overall druggability of 19,450 protein-coding genes and compared our results to DrugnomeAI^9^, a recent druggability prediction model using only tabular features. For stricter and broader definitions of overall druggability, our models had comparable performance to and outperformed DrugnomeAI, respectively (Table S1 and Fig. S4A). Areas under the receiver operating characteristic curve (AUROC) for these definitions were 0.95 (95% CI 0.95–0.95) and 0.94 (95% CI 0.93–0.94), respectively. Predictions were calibrated, with predicted probabilities matching the proportion of druggable genes (Fig. S4B), and there was good performance across gene subsets, including different protein classes and among genes with high PHAROS novelty scores (Fig. S4C). A threshold cutoff of 0.35 maximized the F_1_ score, whereas a 0.5 cutoff yielded a precision of 0.81 and recall of 0.66 (Table 1 and Fig. S4D).

**Table 1:** Recommended cutoffs for each model

Model	Cutoff	*F* score	Precision	Recall
**Gene-level druggability predictions**				
Overall	0.35	*F*_1_ = 0.75	0.74	0.75
Activator	0.18	*F*_1_ = 0.60	0.60	0.60
Inhibitor	0.30	*F*_1_ = 0.68	0.71	0.66
Other	0.17	*F*_1_ = 0.54	0.51	0.57
**Gene-level DOE predictions**				
Activator	0.27	*F*_1_ = 0.69	0.68	0.69
Inhibitor	0.61	*F*_1_ = 0.89	0.85	0.92
Other	0.41	*F*_1_ = 0.72	0.68	0.75
**Gene-disease-specific DOE predictions**				
Activator	0.29	*F*_0.2_ = 0.30	0.31	0.20
Inhibitor	0.69	*F*_0.2_ = 0.72	0.75	0.39
Other	0.30	*F*_0.2_ = 0.31	0.38	0.06

For gene-level predictions, recommended cutoffs are those maximizing the *F*_1_ score, which is the harmonic mean of precision and recall. For gene-disease-specific predictions, recommended cutoffs are those maximizing *F*_0.2_ score, a weighted harmonic mean that heavily favors precision.

**Fig. 3:**
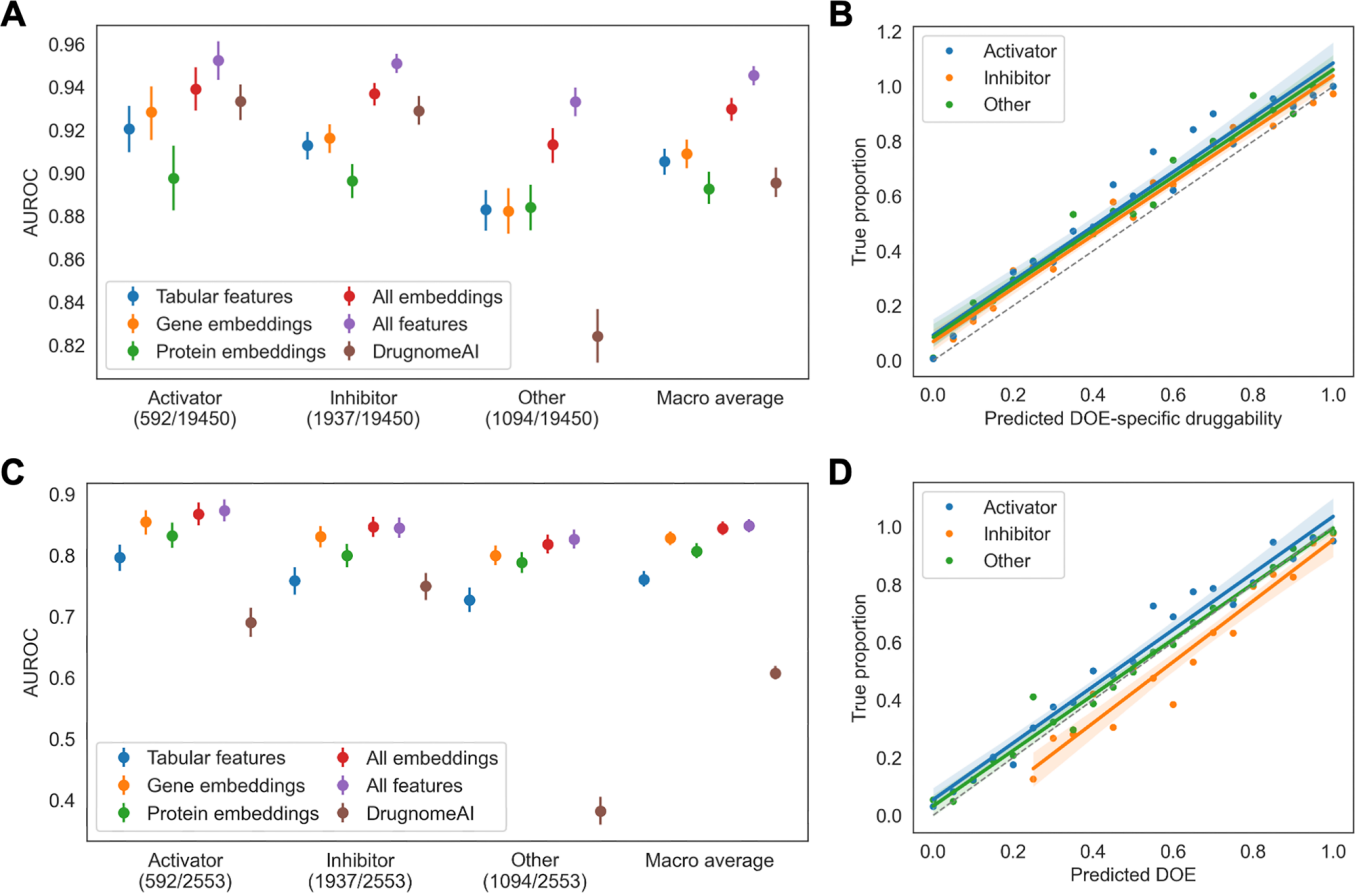
Performance of DOE predictions. **A** AUROC for DOE-specific druggability predictions using different feature sets or DrugnomeAI among 19,450 protein-coding genes. **B** Calibration of DOE-specific druggability predictions. **C** AUROC for predicting DOE among 2553 druggable genes using different feature sets or DrugnomeAI. **D** Calibration of DOE predictions. Shaded regions in **B**, **D** represent 95% confidence intervals for the linear regression lines. We calculated all metrics using holdout predictions. Error bars represent 95% confidence intervals.

We next constructed a DOE-specific druggability model, which substantially outperformed DrugnomeAI and predicted druggability via activator, inhibitor, and other mechanisms with AUROCs of 0.95 (95% CI 0.94–0.96), 0.95 (95% CI 0.95–0.96), and 0.93 (95% CI 0.93–0.94), respectively (Fig. 3A and Table S2). All predictions were calibrated (Fig. 3B), and there was consistent performance across gene subsets (Fig. S5A). DOE-specific and overall druggability predictions were internally consistent: >97% of genes predicted as druggable by a DOE-specific score were also predicted as druggable by the overall score (Fig. S5B–D). Reflecting class imbalances, optimal cutoffs for maximizing F_1_ scores were lower for activator and other mechanism predictions (0.18 and 0.17, respectively) compared to inhibitor predictions (0.30) (Table 1 and Fig. S5E–G).

Both tabular features and embeddings were important across models, with the most important tabular features including research antibody availability, enzyme classification, and mouse knockout phenotypes (Supplementary Data 2, 3). However, for DOE-specific druggability models, 145 features, including constraint metrics, showed opposite direction correlations with importance values for activator and inhibitor predictions (Supplementary Data 3).

Without further training, druggability predictions predicted the clinical trial success and disease relevance of drug targets. First, overall druggability predictions outperformed DrugnomeAI in predicting the progression of drug targets from phase I to phase IV (Fig. S6). Targets with predicted druggability >90th percentile had an OR of 2.58 (95% CI 2.13–3.13) for progressing to phase IV, and this was also true for DOE-specific druggability predictions (Supplementary Data 4). Second, overall druggability predictions were significantly correlated with two target-disease association scores (Mantis-ML and Open Targets) (Fig. S7A–D)^5,8^, suggesting predicted druggable genes are enriched for disease associations.

### Predicting DOE among druggable genes

To separate DOE from druggability and assess the utility of therapeutic modulation, we trained a DOE prediction model among 2553 druggable genes with known drug DOEs. The model using all features predicted activator, inhibitor, and other mechanisms with AUROCs of 0.87 (95% CI 0.86–0.89), 0.85 (95% CI 0.83–0.86), and 0.83 (95% CI 0.81–0.84), respectively (Fig. 3C and Table S3). All predictions were calibrated (Fig. 3D), and there was consistent performance across gene subsets (Fig. S8A).

Activator and inhibitor predictions were weakly negatively correlated (ρ = −0.11, *p* = 3.7 × 10^−8^) (Fig. S8B), whereas other mechanism predictions were positively correlated with activator predictions (ρ = 0.09, *p* = 2.6 × 10^−6^) but negatively correlated with inhibitor predictions (ρ = −0.50, *p* = 8.6 × 10^−165^) (Fig. S8C, D). Among druggable genes, DOE-only predictions were less correlated with overall druggability than DOE-specific druggability predictions (Table S4), suggesting partial isolation of DOE from druggability in these predictions. In feature importance analyses, 206 features had opposite direction correlations with importance values for activator and inhibitor predictions (Supplementary Data 5). Finally, class imbalances resulted in optimal cutoffs for maximizing F_1_ scores being lower for activator and other mechanism predictions (0.27 and 0.41, respectively) compared to inhibitor predictions (0.61) (Table 1 and Fig. S8E–G).

We performed single-sample gene set enrichment analysis to explain each set of predictions. High inhibitor predictions were enriched for cell cycle progression and cell proliferation sets (Fig. S9A and Table S5), reflecting the utility of inhibiting these processes in cancer. High activator predictions were enriched for both pro-inflammatory and anti-inflammatory sets (Fig. S9B), which could inform anti-neoplastic and anti-infective versus immunosuppressive applications, respectively. In contrast, other mechanism predictions were enriched for heterogeneous sets (Fig. S9C). Some of these sets, like angiogenesis and coagulation, contain multiple targets requiring specialized therapeutic modalities, including secreted and structural proteins.

To demonstrate how this model might facilitate novel drug development, we generated DOE predictions for 2179 predicted druggable genes not included in the training set, identifying 199 genes without activator drugs as probable activator targets and 2331 genes without inhibitor drugs as probable inhibitor targets. Manual screening of the top 40 undrugged activator targets showed 33 had an endogenous agonist, 26 had a synthetic agonist, and 23 were associated with treatable phenotypes via activator mechanisms (Supplementary Data 6). For the top 40 undrugged inhibitor targets, 29 had a synthetic antagonist and 27 were associated with treatable phenotypes via inhibitor mechanisms.

We further validated the disease relevance of DOE predictions using Open Targets DOE assessments across 4427 genes and 9217 diseases. Using clinical genetics, genetic associations, and animal models, these assessments determine whether activation or inhibition is therapeutically necessary for each gene-disease pair. Genes with an activator DOE prediction >50th percentile had an OR of 2.00 (95% CI 1.77–2.26) of having an Open Targets activator assessment for any disease, whereas genes with an inhibitor DOE prediction >50th percentile had an OR of 1.25 (95% CI 1.08–1.46) of having an Open Targets inhibitor assessment (Table S6).

### Genetic evidence predicts the direction of effect

**Fig. 4:**
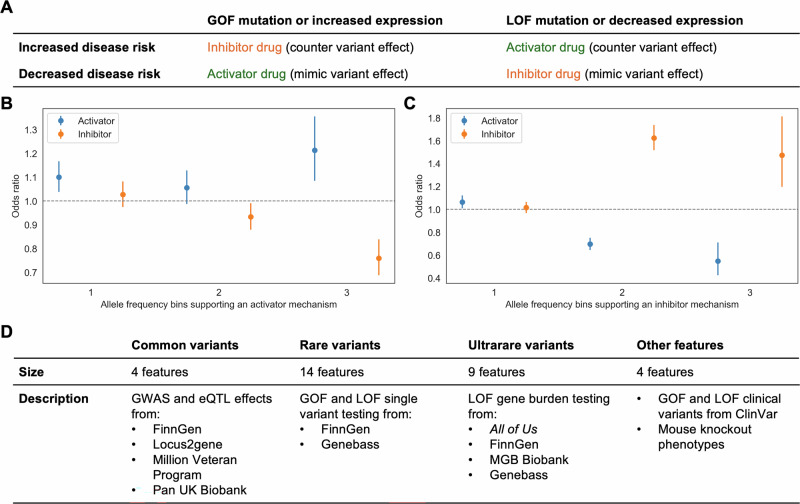
Using human genetics to predict DOE. **A** Framework linking genetic variants to drug mechanisms based on gain-of-function (GOF) or loss-of-function (LOF) effects. **B**, **C** Odds ratios for activator and inhibitor mechanisms based on the number of allele frequency bins (common, rare, ultrarare) supporting each mechanism. **D** Genetic and functional features used to predict gene-disease-specific DOE, categorized by variant type and data source. We calculated all metrics using holdout predictions. Error bars represent 95% confidence intervals.

As genetic variants can mimic drug effects^26^, we next evaluated whether gene-disease-specific evidence could predict DOE. For example, if a GOF variant or increased gene expression protects against disease, an activator drug would likely be beneficial (Fig. 4A). To do so, we analyzed 47,822 gene-disease pairs with indicated drugs, representing 416 diseases and 2029 genes (Supplementary Data 7). Of these pairs, 11,270 (23.6%) had activator mechanisms, 32,064 (67.0%) had inhibitor mechanisms, 8683 (18.2%) had other mechanisms, and 44,079 pairs (92.2%) had only one mechanism.

Only 541 gene-disease pairs were supported by Bonferroni-significant genetic associations, too few for robust analyses. However, at *p* < 0.05, 42,989 of the pairs had supporting associations. Even at this relaxed threshold, the presence of associations from multiple allele frequency categories supporting an activator or inhibitor mechanism yielded significant enrichment of the respective mechanism and depletion of the opposite mechanism (Fig. 4B, C). There was consistent directional support across individual allele frequency bins and increasing support with greater association significance, as indicated by higher −log_10_(*p* values) (Fig. S10A–E). Complementary evidence from Open Targets also predicted DOE, including GOF and LOF clinical variants, mouse knockout phenotypes, and Locus2gene cis-eQTLs (Fig. S10F), but these were available for only 280, 1869, and 280 gene-disease pairs, respectively.

### Predicting gene-disease-specific DOE

**Fig. 5:**
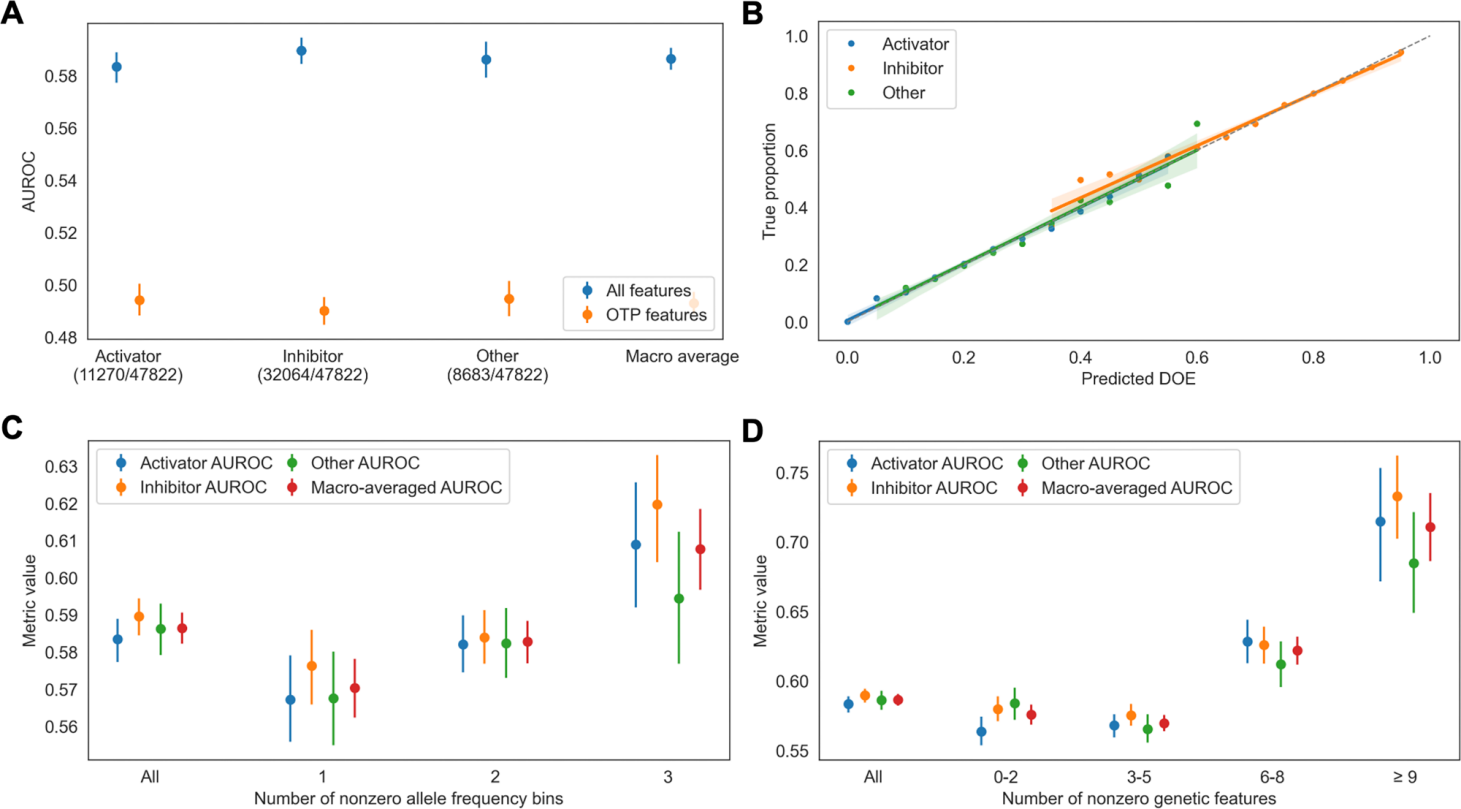
Gene-disease-specific features predict DOE. **A** Areas under the receiver operating characteristic curve (AUROC) for DOE predictions using different feature sets. **B** Calibration of DOE predictions. **C**, **D** AUROC as a function of the number of nonzero allele frequency bins (**C**) or nonzero genetic features (**D**). We calculated all metrics using holdout predictions. Error bars represent 95% confidence intervals.

We used 31 gene-disease-specific genetic features to predict DOE among 47,822 gene-disease pairs (Fig. 4D and Supplementary Data 8). Our model predicted activator, inhibitor, and other mechanisms with AUROCs of 0.58 (95% CI 0.58–0.59), 0.59 (95% CI 0.58–0.59), and 0.59 (95% CI 0.58–0.59), respectively (Fig. 5A and Supplementary Data 9), and all predictions were calibrated (Fig. 5B). Importantly, model performance increased with both the number of nonzero allele frequency bins and the number of nonzero genetic features (Fig. 5C, D). Among 1075 gene-disease pairs where ≥9/31 features were nonzero, there was a macro-averaged AUROC of 0.71 (95% CI 0.69–0.74). Our model also significantly outperformed models trained using only OTP features, which had macro-averaged AUROCs of 0.49 (95% CI 0.49–0.50) among all gene-disease pairs and 0.56 (95% CI 0.54–0.58) among 2237 pairs where at least one OTP feature was nonzero (Supplementary Data 9).

Given the modest model performance, we recommend prioritizing precision over recall and using higher cutoffs. Optimal cutoffs for maximizing *F*_0.2_ scores were 0.29, 0.69, and 0.30 for activator, inhibitor, and other mechanisms, yielding precisions of 0.31, 0.75, and 0.38, respectively (Table 1 and Fig. S11A–C).

The most important features were primarily common variant associations (Supplementary Data 10), likely due to their greater availability. However, when considering only nonzero feature values, rare variant and gene-burden features became more important, with the top three features being GOF rare variant features. For activator and inhibitor predictions, there were directionally consistent correlations between feature values and importance values. For example, GOF rare variant features, encoded as sign_beta _× −log_10_(*p* value), were positively correlated with inhibitor predictions and negatively correlated with activator predictions, whereas the opposite was true for LOF rare variant features. In contrast, correlations for other mechanism predictions had varying directions, consistent with such drugs having either, neither or mixed activator and inhibitor properties.

To demonstrate how these predictions can support novel drug development, we analyzed 56,089 gene-disease pairs not included in our training set that met three criteria: (1) strong target-disease associations from Mantis-ML or Open Targets^5,8^, (2) the gene is known or predicted to be druggable, and (3) there are no existing drug indications. We applied the first criterion because gene-disease-specific DOE predictions solely represent DOE and do not indicate mechanistic or therapeutic importance (Table S7). Several high-scoring activator and inhibitor predictions were supported directly or indirectly by preclinical evidence (Table S8), such as *CFH* activation/replacement for retinal disorders^27^, *MC4R* activation for type 2 diabetes^28^, *TERT* activation/replacement for interstitial lung disease^29^, *LRP3* inhibition for erythematous conditions^30^, and *KIT* inhibition for polycystic kidney disease^31^.

### Comparing gene-level and gene-disease-specific DOE predictions

We next compared gene-level to gene-disease-specific DOE predictions for gene-disease-specific DOE prediction among 47,822 gene-disease pairs, with the two predictions being weakly correlated (Fig. S12A–C). Despite being disease-agnostic, gene-level DOE predictions significantly outperformed gene-disease-specific DOE predictions (Fig. S13A), which remained true when restricting to 404 genes targeted by both activator and inhibitor drugs (Fig. S13B), and when analyzing top percentiles of both scores (Supplementary Data 11). This is likely because most gene-disease pairs lacked sufficient genetic association evidence for confident predictions, whereas gene-level features had low missingness rates (Supplementary Data 1).

Nevertheless, gene-disease-specific DOE predictions remain important for three reasons. First, they provide disease-specific context for the ~3% of druggable genes with high gene-level predictions for both activation and inhibition, such as *ADRB1* and *HTR1A* (Fig. S8B and Table S9). Second, the performance gap between gene-level and gene-disease-specific DOE predictions decreases as the number of nonzero genetic features increases (Supplementary Data 9). Third, at matched percentile thresholds, gene-disease-specific predictions were more consistently associated with the clinical trial success of target-disease-mechanism triplets (Supplementary Data 4). Overall, gene-level and gene-disease-specific predictions are complementary: both were significantly associated with DOE in a multivariable logistic regression (Fig. S13C), and intersecting the two predictions for each DOE yielded greater enrichment for gene-disease pairs with the respective DOE (Table S10).

## Discussion

We present a framework to refine target selection and modulation using three DOE prediction models (Fig. S1). First, the gene-level DOE-specific druggability model aims to expand the scope of druggable targets for each DOE, especially for activation. Second, the gene-level DOE model predicts DOE independent of druggability and could suggest the therapeutic utility of modulating a target in a certain direction across diseases. Third, the gene-disease-specific DOE model predicts the correct DOE for each gene-disease pair. The first two models, along with the genetic and functional differences we observed between activator and inhibitor drug targets, suggest that DOE can be considered both at the gene level and in a gene-disease-specific manner. Importantly, DOE predictions were associated with clinical trial success, suggesting potential value in de-risking drug targets.

Our findings may offer actionable insights for drug development. Predictions from all three models are calibrated such that the outputs represent the true proportion of positives in each class, but we also provide binary cutoffs that maximize F scores (Table 1). Gene-level models are disease-agonistic and prioritize targets where developing novel activator or inhibitor drugs is likely to be both feasible and therapeutically useful (e.g., *GRM8* agonism for neuroprotection)^32^. Moreover, there are substantially more known inhibitor targets (75.9% of druggable genes in our dataset) compared to activator targets (23.2%), with activator targets concentrated in limited classes like G protein-coupled receptors. Combining our models with emerging structure-based approaches could help address this imbalance, particularly for mechanisms like enzyme activation, where allosteric drug design remains difficult^33,34^. For gene-disease DOE prediction, gene-level models generally outperformed gene-disease-specific models, but intersecting the two predictions may be useful when disease-specific context is required or multiple lines of genetic evidence are available. We caution that none of these models indicates whether a gene is an effective target for a given disease, and gene-disease causality should be separately established using human genetics in conjunction with animal models and experimental evidence^4,35^. Therefore, DOE predictions should be considered alongside target-disease association scores like Mantis-ML^5^, Open Targets^8^, or GPS^6^. Overall, although experimental confirmation is still needed, a confident DOE determination early in the pipeline, either alongside or after target selection, could facilitate drug development.

This study has several limitations. First, we relied on existing druggable genes and known drug mechanisms, which may bias predictions toward prevalent drug modalities like small molecules. Although we incorporated data from five drug sources to improve coverage, emerging therapeutic strategies like gene therapies, mRNA-based treatments, and PROTACs may alter the landscape of druggability and DOE feasibility. Second, while embeddings enable the model to learn druggability and DOE patterns independently of human labels, they reduce interpretability compared to tabular features. Third, the clinical trial outcome data we use may underrepresent failed clinical trials. Fourth, we defined diseases for gene-disease models primarily using three-character ICD-10 codes to maximize compatibility with existing summary statistics. However, these codes have variable sensitivity and specificity for capturing disease processes and may have suboptimal granularity. Fifth, we relied on nominally significant genetic associations and predicted GOF and LOF variant classifications for gene-disease-specific analyses, which could have resulted in spurious predictions. Sixth, we do not prospectively validate our models; future evidence from drug development efforts, particularly involving currently undrugged genes, will be necessary to determine whether real-world outcomes are consistent with our predictions.

There are also opportunities to extend this work. Our models do not account for the degree of target modulation due to limited training data, but distinguishing between effective modulation and toxicity is critical since many targets have narrow therapeutic windows. Additionally, numerous factors besides DOE contribute to failed drug development, including insufficient causal gene-disease evidence, issues with the drug itself, and suboptimal clinical trial design. These downstream challenges could benefit from complementary computational strategies.

In summary, we developed and validated three complementary models for predicting therapeutic DOE. We demonstrate that DOE can be predicted both as a disease-agnostic property using gene-level features and, when sufficient data is available, as a disease-specific property using human genetics evidence. Combining these approaches guides therapeutic modulation strategies and can help accelerate and de-risk drug development.

## Methods

### Ethics approval

All datasets used in this study were publicly available and de-identified. No ethics approval was necessary.

### Obtaining drug mechanisms and indications

We compiled a comprehensive dataset of drug-target interactions and mechanisms of action by integrating data from multiple sources, including ChEMBL (version 35)^36^, Open Targets Platform (OTP; release 2024.09)^8^, DrugBank (version 5.1.13)^37^, Guide to Pharmacology (release 2024.4)^38^, and Santos et al.’s manually curated map^39^. From ChEMBL, we included only compounds included in the DRUG_INDICATION and DRUG_MECHANISM tables, which include approved and clinical candidate drugs. From the Guide to Pharmacology, we included only approved drugs, as there was no filter for non-approved clinical candidate drugs.

To create a unified drug mechanism dataset, we first compiled conversions between different identifiers (e.g., ChEMBL IDs, DrugBank IDs, Guide to Pharmacology IDs, PubChem CIDs, and generic drug names) using the PubChem Identifier Exchange Service as well as conversion tables provided by each data source. We then unified drug representations across sources using a disjoint-set approach, assigning a unique ID to each drug entity based on shared identifiers. For each drug, we then merged mechanisms of action from the different sources and classified them as activator, inhibitor, or other (Table S11). This ultimately yielded 22,039 drug-gene pairs representing 7341 drugs and 2553 genes. For 549 of 22,039 drug-gene pairs with discrepant mechanisms between sources, we prioritized annotations from ChEMBL and DrugBank over other sources, as well as annotations of either activator or inhibitor mechanisms over other mechanisms, which resolved all discrepancies.

For gene-disease-specific models, we obtained drug indications from OTP and from the orphan drug databases of the Food and Drug Administration and the European Medicines Agency. We mapped OTP drug indications to ICD-10 codes using OTP-supplied conversion files and the UMLS Metathesaurus (release 2024AA) and merged this with our drug mechanism dataset via ChEMBL IDs. For orphan drug data, we mapped drug names to ChEMBL IDs using ChEMBL molecule synonyms and manually mapped drug indications to ICD-10 codes (Supplementary Data 12).

### Features for gene-level models

For all gene-level models, we included 425 features: 41 tabular features, gene embedding vectors with 256 dimensions, and protein embedding vectors with 128 dimensions. Descriptions of the 41 tabular features are available in Table S1. Briefly, they include the number of research monoclonal antibodies from Antibodypedia^40^, target prioritization data from Open Targets^8^, oncogene and tumor suppressor gene assignments from OncoKB^41^, protein localization and function from The Human Protein Atlas^42^, constraint data from gnomAD (version 4.1)^43^, 3089 manually curated GOF variants involved in 1299 diseases from GoFCards^44^, gene involvement in autosomal dominant and recessive diseases from online Mendelian inheritance in man (OMIM)^45^, common essential and strongly selective designations from DepMap^46^, haploinsufficiency from ClinGen^47^, predicted disease involvement via GOF, LOF, or dominant negative mechanisms^18^, predicted haploinsufficiency and triplosensitivity^16^, tau index of gene tissue specificity from GTEx (version 10)^48^, and pocket predictions on AlphaFold 2 structures using fpocket (version 4.2.2)^49,50^.

We obtained gene embeddings from GenePT^21^, which employs OpenAI’s text-embedding-3-large model to encode National Center for Biotechnology Information textual gene descriptions as 3072-dimensional vectors^51^. These descriptions describe the characteristics, functions, and disease associations of each gene but do not contain drug indications or mechanisms. Because text-embedding-3-large was trained using Matryoshka Representation Learning, where earlier embedding dimensions contain more significant information (Fig. S14A), it is possible to use truncated vectors without substantial loss of meaning. To balance feature count and model performance, we assessed different embedding dimensionalities and selected 256 dimensions as optimal (Fig. S14B).

We obtained protein embeddings from UniProt, which used the prottrans_t5_xl_u50 model^52^, a T5 Transformer-based protein language model, to represent amino acid sequences as 1024-dimensional vectors. These vectors are available for all proteins with fewer than 12,000 residues. We used principal component analysis from scikit-learn (Python package version 1.5.2) to reduce the dimensionality of these vectors and found that 128 dimensions was optimal in terms of both information loss and model performance (Fig. S14C, D).

To facilitate interpretation of gene and protein embeddings, we used umap-learn (Python package version 0.5.7) to generate two-dimensional representations using n_neighbors = 15 and min_dist = 0.1. Plotting these two dimensions showed that druggable genes, as well as genes targeted by activator, inhibitor, and other mechanism drugs, formed distinct clusters (Fig. S15A, B).

### Constructing machine learning models

We trained all machine learning models using XGBoost (Python package version 2.1.3) with a nested eightfold cross-validation framework. In the outer loop, we randomly split the dataset into eightfolds, using one as a holdout test set while training on the remaining data. Within each outer fold, we performed an inner eightfold cross-validation, where onefold served as a validation set and the model was trained on the remaining inner folds. After training, each inner fold model generated predictions on the outer holdout set, and we averaged these predictions across inner folds. Repeating this process for all outer folds ensured a robust evaluation. To prevent overfitting, we used default XGBoost hyperparameters except for min_child_weight = 10 to prevent overly complex splits. We also enabled early stopping after ten rounds of no improvement. To assess feature importance, we enabled SHapley Additive exPlanations (SHAP; Python package version 0.46.0) when generating holdout predictions.

As a sensitivity analysis comparing model types, we compared XGBoost to convolutional neural networks (CNN) and logistic regression (LR) and found that XGBoost outperformed both in predicting overall and DOE-specific druggability (Table S12). We trained CNNs using TensorFlow (Python package version 2.18.0) within the same nested cross-validation framework. The architecture included three convolutional layers with ReLU activation. The first two layers had 64 filters with a kernel size of 3, followed by batch normalization and max-pooling. The third convolutional layer used 128 filters, followed by another max-pooling step. We then flattened the output, passed it through a fully connected layer with 64 neurons and ReLU activation, and applied dropout (0.2) before the final dense layer with a sigmoid activation function. We trained the model using the Adam optimizer (learning rate = 0.001) and binary cross-entropy loss. We applied early stopping with a patience of ten epochs and restored the best model based on validation loss. For LR models, we used scikit-learn and used an unnested eightfold cross-validation framework, as LR does not support early stopping. In each fold, we trained on seven folds while reserving one for holdout testing.

Among 24 of the 41 gene-level features with missing values, there was a median missingness rate of 7.1% (range = 0.9–21.0%) (Table S1). XGBoost models support missing values and do not benefit from feature normalization. For CNN and LR models, we imputed missing values with either the median value (continuous features) or zeros (binary features) and normalized values during each fold using mean and variance parameters from the training set.

To assess whether our models generalize to undercharacterized targets, we performed a second sensitivity analysis where we trained models on well-characterized genes (low PHAROS novelty scores) and evaluated them on less-characterized genes (high PHAROS novelty scores). PHAROS novelty scores represent the relative abundance of publication mentions of each target^53^. For gene-level overall druggability, gene-level DOE, and gene-disease-specific DOE models, we trained and validated on the bottom 7/8 of genes ranked by PHAROS novelty scores using eightfold cross-validation, and generated holdout predictions for the top 1/8. For DOE-specific druggability models, we used the bottom 1/2 for training and validation and the top 1/2 for holdout testing, due to insufficient positive examples in smaller subsets. All models achieved AUROCs comparable to those trained on random splits (Supplementary Data 13). Combined with subset analyses showing strong performance on novel genes (Figs. S4C, S5A, S8A), these results suggest our models generalize to genes with limited prior annotation.

We compared gene-level models to DrugnomeAI, which used XGBoost with 324 features to predict druggability^9^. DrugnomeAI is available for several different definitions of druggability, including definitions based on Pharos (Tclin, Tchem, Tbio) and Triage (Tier 1, Tier 2, Tier 3) resources^53,54^. We compared these models and selected Tclin + Tchem as the best-performing model (Table S13).

### Clinical trial success

We assessed the ability of druggability and DOE predictions to predict clinical trial success using Open Targets data (target-disease evidence by source: ChEMBL). For each phase transition from X to Y, we classified drug targets (for overall druggability), target-DOE pairs (for DOE-specific druggability), and target-DOE-disease triplets (for gene-disease-specific DOE) as successes if any associated drug reached phase Y. We labeled them as failures if they had drugs that progressed to at least phase X but did not advance beyond phase Y-1 and were no longer in active development. We classified the following statuses as active development: recruiting; active, not recruiting; not yet recruiting; enrolling by invitation. For target-DOE pairs and target-DOE-disease triplets, we performed separate analyses for each DOE.

### Target-disease association scores

We used target-disease association scores from Mantis-ML and Open Targets to determine the disease relevance of gene-level druggability and DOE predictions, and to select gene-disease pairs with strong association evidence for gene-disease-specific DOE predictions. Mantis-ML uses a graph neural network with both tabular features and a knowledge graph to rank gene-disease pairs, with data sources including GWAS, OMIM, and biological processes. Open Targets provides individual scores for each of 23 evidence sources that represent evidence strength; these are then unified into a single score via a weighted harmonic sum. Open Targets additionally provides DOE assessments for seven evidence sources (Locus2gene, gene burden, ClinVar germline, ClinVar somatic, Gene2Phenotype, Orphanet, and IMPC)^8,55–59^. For both scores, we used the maximum score per gene across all diseases to validate gene-level druggability predictions. For the Open Targets score, we used the maximum score per gene-DOE pair across all diseases to validate gene-level DOE predictions.

### Disease definitions

We defined diseases using three-character ICD-10 codes to maximize phenotype compatibility with public genetic association datasets. Adapting definitions from the World Health Organization, we excluded ICD-10 codes that represented communicable, maternal, perinatal, and nutritional conditions, injuries, or ill-defined diseases^60^. We also included 15 custom phenotypes that were included in Genebass that represented either multiple three-character ICD-10 codes (e.g., stroke) or required more granularity than three characters (e.g., celiac disease) (Supplementary Data 14). Ultimately, we included 547 diseases (Supplementary Data 7), of which 416 had at least one indicated drug and were included in our training set.

We downloaded pre-computed genetic association testing results from FinnGen (Freeze 12)^61^, Genebass^62^, Million Veteran Program^63^, Pan-UK Biobank^64^, and rare variant testing by Jurgens et al., representing a meta-analysis of pan-ancestry gene burden testing in *All of Us*, the Mass General Brigham Biobank, and the UK Biobank^65^. For FinnGen, we included all phenotypes representing a single three-character ICD-10 code and manually mapped phenotypes for custom codes. For Genebass, we included icd_first_occurrence and icd10 phenotypes as well as custom codes. The Million Veteran Program, Pan-UK Biobank, and Jurgens et al.’s meta-analysis all used phecode1.2 to define phenotypes; we included all phecodes representing a single ICD-10 code and manually mapped phecodes to custom codes (Supplementary Data 14). From the Pan-UK Biobank, we additionally included ICD-10 traits. A list of all traits included from each dataset is available in Supplementary Data 15.

### Features for gene-disease-specific models

Descriptions of the 31 features we used for gene-disease-specific models are available in Supplementary Data 8. Across all datasets, we included only genetic associations with *p* < 0.05 and encoded these associations as −log_10_(*p* values) to allow models to assign greater weight to more significant associations. Feature importance analyses showed this was indeed the case (Supplementary Data 10), where feature values were significantly correlated with importance values. For gene-disease pairs tested in a dataset but lacking associations with *p* < 0.05, we assigned a value of 0. If a gene-disease pair was not tested in a particular dataset, we left the value as missing.

For common variant associations from FinnGen, Million Veteran Program, and Pan-UK Biobank, we intersected variant-disease associations with significant eQTL associations from the Genotype-Tissue Expression (GTEx) project (version 10). If a single variant had eQTLs corresponding to multiple genes, we kept the eQTL corresponding to the closest gene to the variant. We then grouped variants by whether the sign of the effect beta and the sign of the eQTL beta were the same (predicting an inhibitor mechanism) or opposite (predicting an activator mechanism), and retained the most significant association in each gene. We used a similar approach to incorporate Locus2gene evidence, but used eQTL associations provided by Open Targets, calculated a normalized harmonic sum of Locus2gene scores across all variants with the same predicted mechanism in each gene, and retained the larger sum. This yielded four common variant features (Supplementary Data 8).

For rare coding variants from FinnGen and Genebass, as well as clinical variants from ClinVar, we first performed variant annotation using Ensembl variant effect predictor (VEP; release 112). For all stop-gained, splice site-disrupting, and frameshift variants, we then performed LOFTEE (version 1.0.4, GRCh38 branch) to classify these variants as either high-confidence or low-confidence LOF. For remaining missense variants, we classified them as LOF, GOF, or other using predictions from LoGoFunc, an ensemble model leveraging gene-, protein-, and variant-level features^19^. We supplemented these predictions with 3089 manually annotated GOF variants from GoFCards^44^. For all missense variants, we additionally calculated a missense Score to quantify the deleteriousness of the variant using the dbNSFP (version 4.9a) plugin for Ensembl VEP, similar to prior studies^65,66^. Briefly, this score ranges from 0 to 1 and represents the proportion of 30 algorithms that determine a variant to be deleterious; we kept scores only if at least 8 of the 30 returned a prediction. The 30 algorithms included 21 qualitative tools (SIFT, SIFT4G, Polyphen2_HDIV, Polyphen2_HVAR, LRT, MutationTaster, FATHMM, PROVEAN, MetaSVM, MetaLR, M-CAP, PrimateAI, DEOGEN2, BayesDel_addAF, BayesDel_noAF, ClinPred, LIST-S2, fathmm-MKL_coding, fathmm-XF_coding, MutationAssessor, and Aloft), where we defined deleterious as H for MutationAssessor, R or D for Aloft, and D for all other tools, as well as nine quantitative tools (VEST4, REVEL, MutPred, MVP, MPC, DANN, CADD_raw, Eigen-raw_coding, and Eigen-PC-raw_coding), where we defined deleterious as a rank score >0.9. Using both GOF/LOF assignments and missense score predictions, we created 14 rare variant categories as features (Supplementary Data 8). We encoded features as sign_beta_ × −log_10_(*p* value) for the most significant variant in each category.

For gene burden testing of rare and ultrarare coding variants, which we obtained from FinnGen, Genebass, and a meta-analysis by Jurgens et al., we created nine features (Supplementary Data 8). We encoded features as sign_beta_ × −log_10_(*p* value).

### Statistical analyses

We performed all analyses using Python 3.12. We evaluated model performance by aggregating holdout predictions across the eight outer folds from nested cross-validation and computing metrics over the entire dataset using scikit-learn. To estimate uncertainty, we calculated the mean and 95% confidence interval for each metric using 1000 iterations of the reverse percentile bootstrap. For models with multiple classes (e.g., activator, inhibitor, and other), we additionally calculated macro-averaged and micro-averaged metrics. Macro-averaging computes the metric independently for each class and then takes the average, treating all classes equally, whereas micro-averaging aggregates predictions across classes to compute an overall metric, giving more weight to classes with more samples. We performed statistical tests and calculated correlations using scipy (Python package version 1.14.1). All tests were two-sided, and we considered *p* < 0.05 significant. We performed logistic regressions to calculate odds ratios using statsmodels (Python package version 0.14.4). For logistic regression, we generally compared genes or gene-disease pairs above a percentile threshold or cutoff to those below; additional details are available in the relevant Supplementary Tables.

## Supplementary information

Supplementary information

Supplementary data 1

Supplementary data 2

Supplementary data 3

Supplementary data 4

Supplementary data 5

Supplementary data 6

Supplementary data 7

Supplementary data 8

Supplementary data 9

Supplementary data 10

Supplementary data 11

Supplementary data 12

Supplementary data 13

Supplementary data 14

Supplementary data 15

## Data Availability

Data, including predictions from all models, are available at https://github.com/robchiral/DOE-prediction and 10.5281/zenodo.15001635. Other data sources used in this study are publicly available, including ChEMBL (https://chembl.gitbook.io/chembl-interface-documentation/downloads), DrugBank (https://go.drugbank.com/releases/latest), FinnGen (https://www.finngen.fi/en/access_results), Genebass (https://app.genebass.org/downloads), gnomAD constraint data (https://gnomad.broadinstitute.org/data#v4-constraint), Million Veteran Program (https://ftp.ncbi.nlm.nih.gov/dbgap/studies/phs002453), Pan-UK Biobank (https://pan.ukbb.broadinstitute.org/downloads), Open Targets (https://platform.opentargets.org/downloads), and the UMLS Metathesaurus (https://www.nlm.nih.gov/research/umls/licensedcontent/umlsknowledgesources.html).

## Code availability

Code is available at https://github.com/robchiral/DOE-prediction.
