# Supplementary material for: Genetic evidence informs the direction of therapeutic modulation in drug development
Source: NPJ Drug Discov. 2025 Oct 1;2:24. doi: 10.1038/s44386-025-00027-0 (PMC12488494; doi:10.1038/s44386-025-00027-0)
Supplement: Supplementary file 1 — Supplementary information [file 44386_2025_27_MOESM1_ESM.pdf]

Supplementary information for “Genetic evidence informs the direction of therapeutic modulation in drug development”

Contents

Supplementary figures

Figure S1: Overview of gene-level and gene-disease models

Figure S2: Additional characteristics of drugs and targets

Figure S3: Predicted haploinsufficiency and triplosensitivity across drug target categories

Figure S4: Performance metrics for gene-level druggability predictions

Figure S5: Performance metrics for DOE-specific druggability predictions

Figure S6: Overall druggability predictions also predict clinical trial success

Figure S7: Correlation of predicted druggability with target-disease association scores

Figure S8: Performance metrics for DOE predictions

Figure S9: Gene set enrichment analysis of DOE predictions

Figure S10: Genetic evidence supports DOE predictions across allele frequency bins and data sources

Figure S11: Performance metrics for gene-disease-specific DOE predictions

Figure S12: Correlation between gene-level and gene-disease-specific DOE predictions

Figure S13: Comparison of gene-level and gene-disease-specific DOE models

Figure S14: Optimization of gene and protein embeddings

Figure S15: UMAP visualization of gene and protein embeddings

Supplementary tables

Table S1: Metrics for predicting overall druggability

Table S2: Metrics for predicting DOE-specific druggability

Table S3: Metrics for predicting DOE

Table S4: Correlations between different predictions

Table S5: Single sample gene set enrichment with DOE predictions

Table S6: Disease relevance of gene-level DOE predictions

Table S7: Correlations of gene-disease DOE predictions with association scores

Table S8: Examples of novel targets with confident gene-disease-specific DOE predictions

Table S9: Genes with simultaneously high (> 0.9) activator and inhibitor gene-level DOE predictions

Table S10: Odds ratios for gene-disease pairs supported by gene-level and/or gene-disease-specific DOE predictions

Table S11: Classification of drug mechanisms

Table S12: Comparison of model architectures for predicting overall and DOE-specific druggability

Table S13: Comparison of DrugnomeAI models for predicting overall druggability

Supplementary data

Supplementary Data 1: Tabular features used in gene-level models

Supplementary Data 2: Feature importances for all-features model for predicting overall druggability

Supplementary Data 3: Feature importances for all-features model for predicting DOE-specific druggability

Supplementary Data 4: Metrics for predicting clinical trial success

Supplementary Data 5: Feature importances for all-features model for predicting DOE

Supplementary Data 6: Manual screening of top activator or inhibitor predictions without indicated drugs

Supplementary Data 7: Diseases included for gene-disease-specific analyses

Supplementary Data 8: Features used in gene-disease-specific models

Supplementary Data 9: Metrics for predicting gene-disease-specific DOE

Supplementary Data 10: Feature importances for all-features model for predicting gene-disease-specific DOE

Supplementary Data 11: Comparing gene-level and gene-disease-specific DOE models for gene-disease-specific DOE prediction

Supplementary Data 12: Orphan drug indications mapped to ICD-10 codes

Supplementary Data 13: Performance of models trained with non-random splits based on PHAROS novelty scores

Supplementary Data 14: Definitions of custom phenotypes

Supplementary Data 15: Diseases included in each dataset

Figure S1: Overview of gene-level and gene-disease models

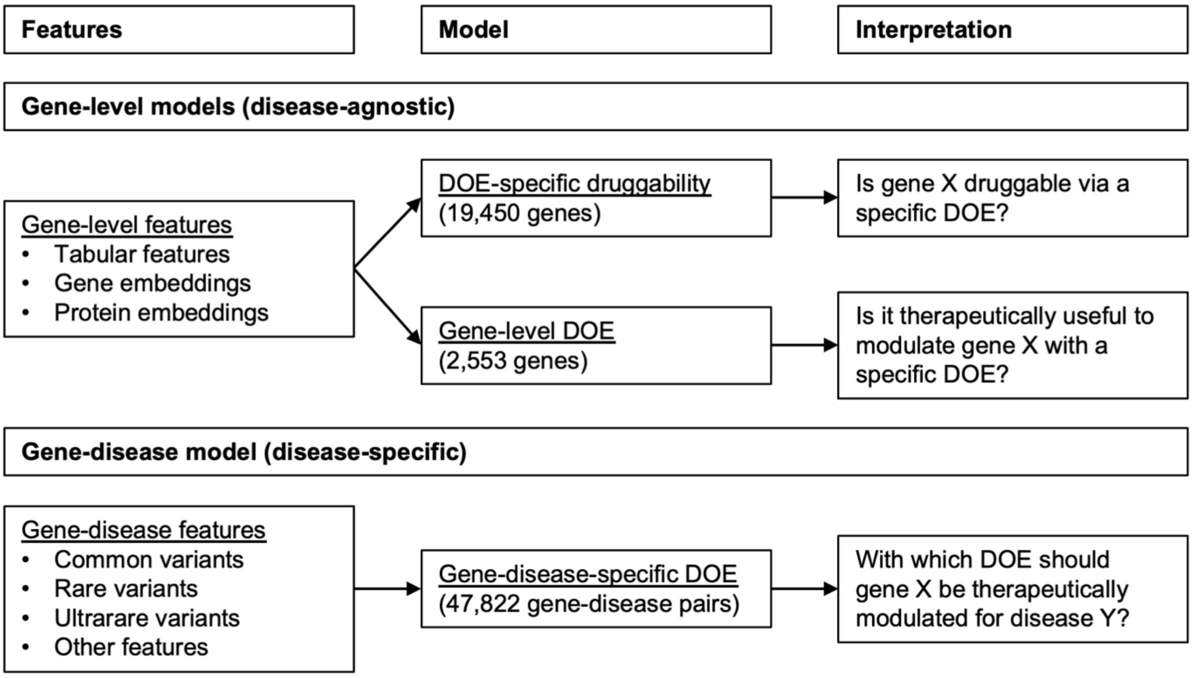

Figure S2: Additional characteristics of drugs and targets

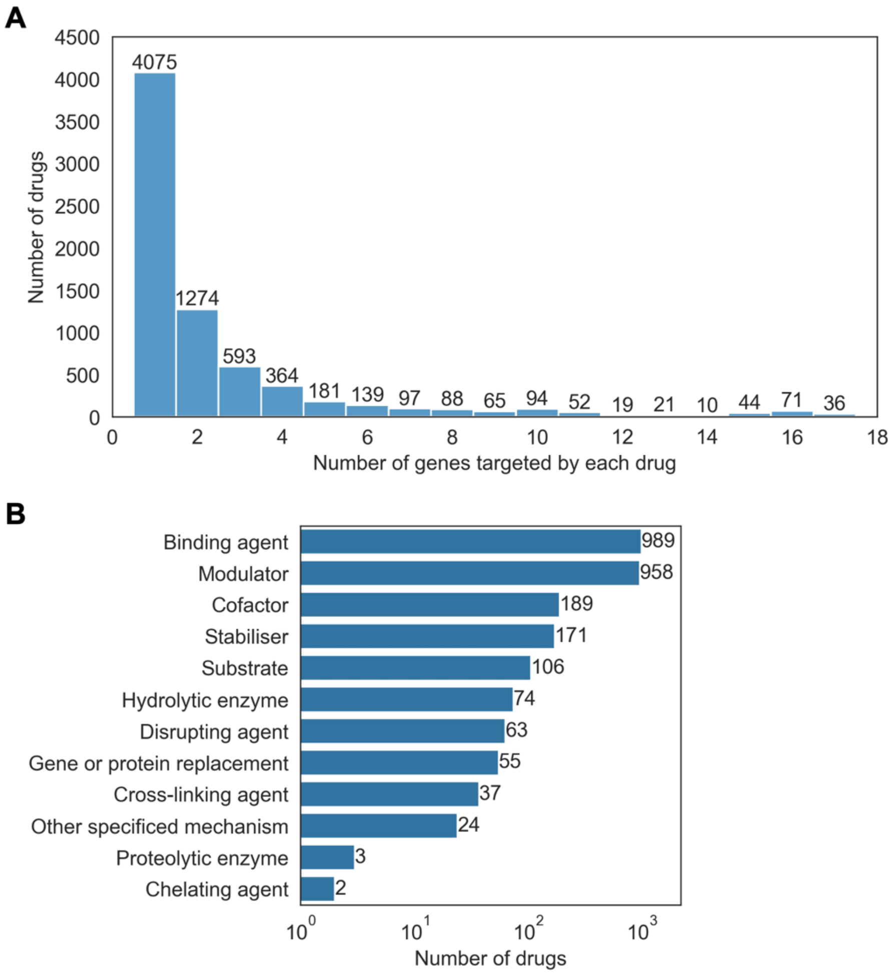

(A) Histogram showing the number of genes targeted by each drug. **(B)** Drug mechanisms classified as other. The x-axis is in $\log_{10}$ scale.

Figure S3: Predicted haploinsufficiency and triplosensitivity across drug target categories

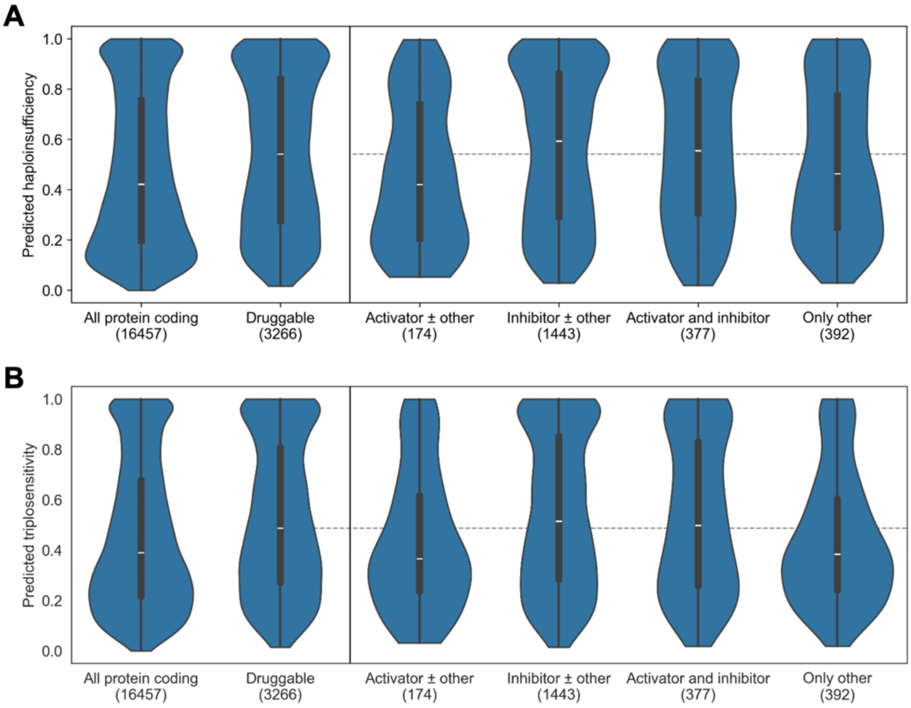

Violin plots of predicted haploinsufficiency (**A**) and predicted triplosensitivity **(B)** for different gene categories. Each gene can be targeted by drugs with different DOEs; for example, “Activator ± other” indicates genes targeted by activator drugs, some of which may also be targeted by drugs with other mechanisms. Dashed lines indicate the median values for druggable genes. Numbers in parentheses indicate the number of genes in each category with non-missing values.

Figure S4: Performance metrics for gene-level druggability predictions

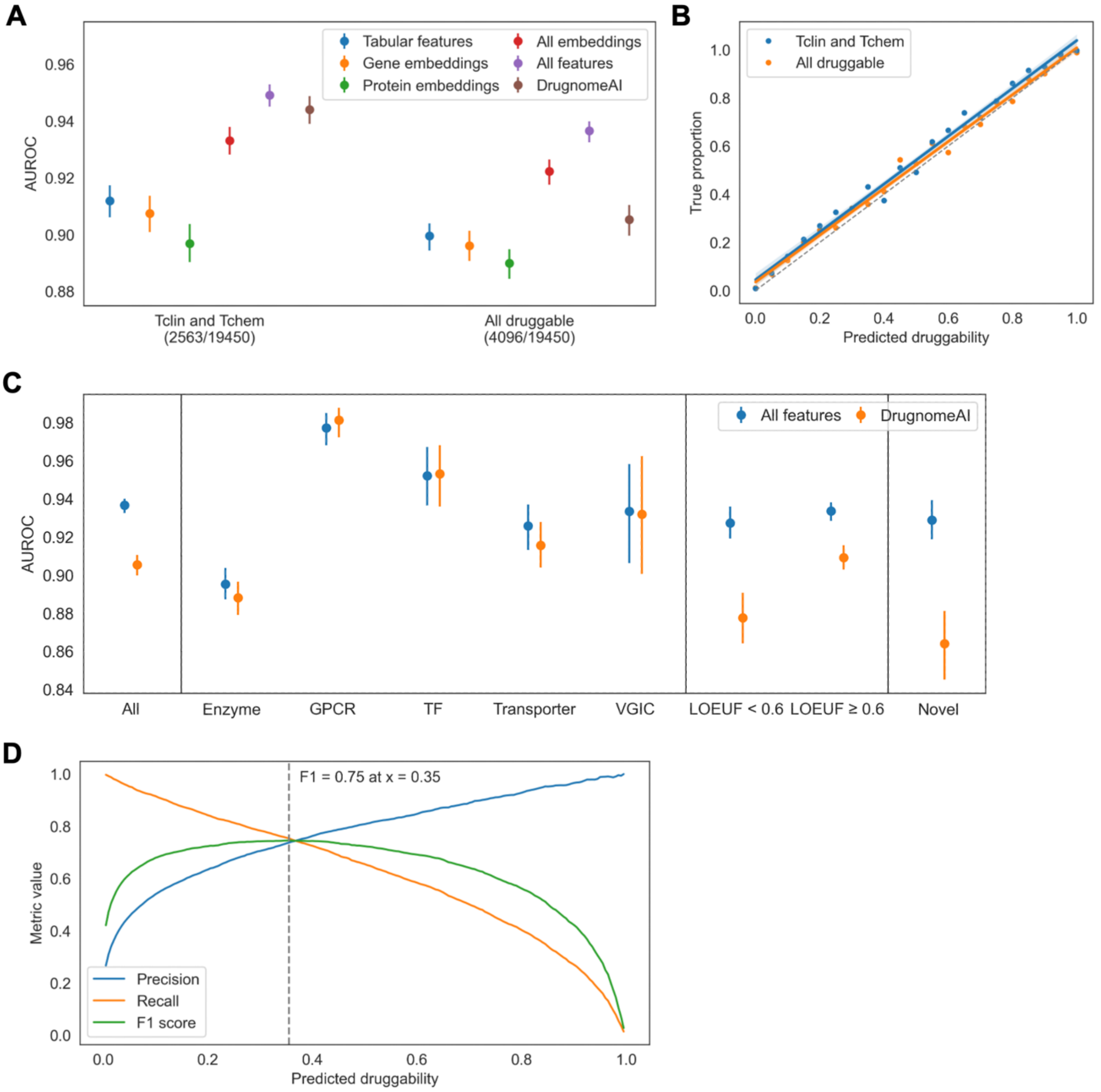

(A) Areas under the receiver operating characteristic curve (AUROC) for druggability predictions using different feature sets or DrugnomeAI. Left: stricter druggability definition (Tclin + Tchem). Right: expanded druggability definition. **(B)** Calibration of druggability predictions. **(C)** AUROCs for druggability predictions across different gene categories, including all protein-coding genes, enzymes, G protein-coupled receptors (GPCRs), transcription factors (TFs), transporters, voltage-gated ion channels (VGICs), constraint groups (LOEUF < 0.6 versus LOEUF ≥ 0.6), and genes with above-median PHAROS novelty scores (score > 0.038). **(D)** Precision, recall, and F_1 score as a function of predicted druggability, with the dashed line indicating the cutoff yielding a maximum F_1 score. Error bars represent 95% confidence intervals. All plots represent holdout performance among 19,450 protein-coding genes.

Figure S5: Performance metrics for DOE-specific druggability predictions

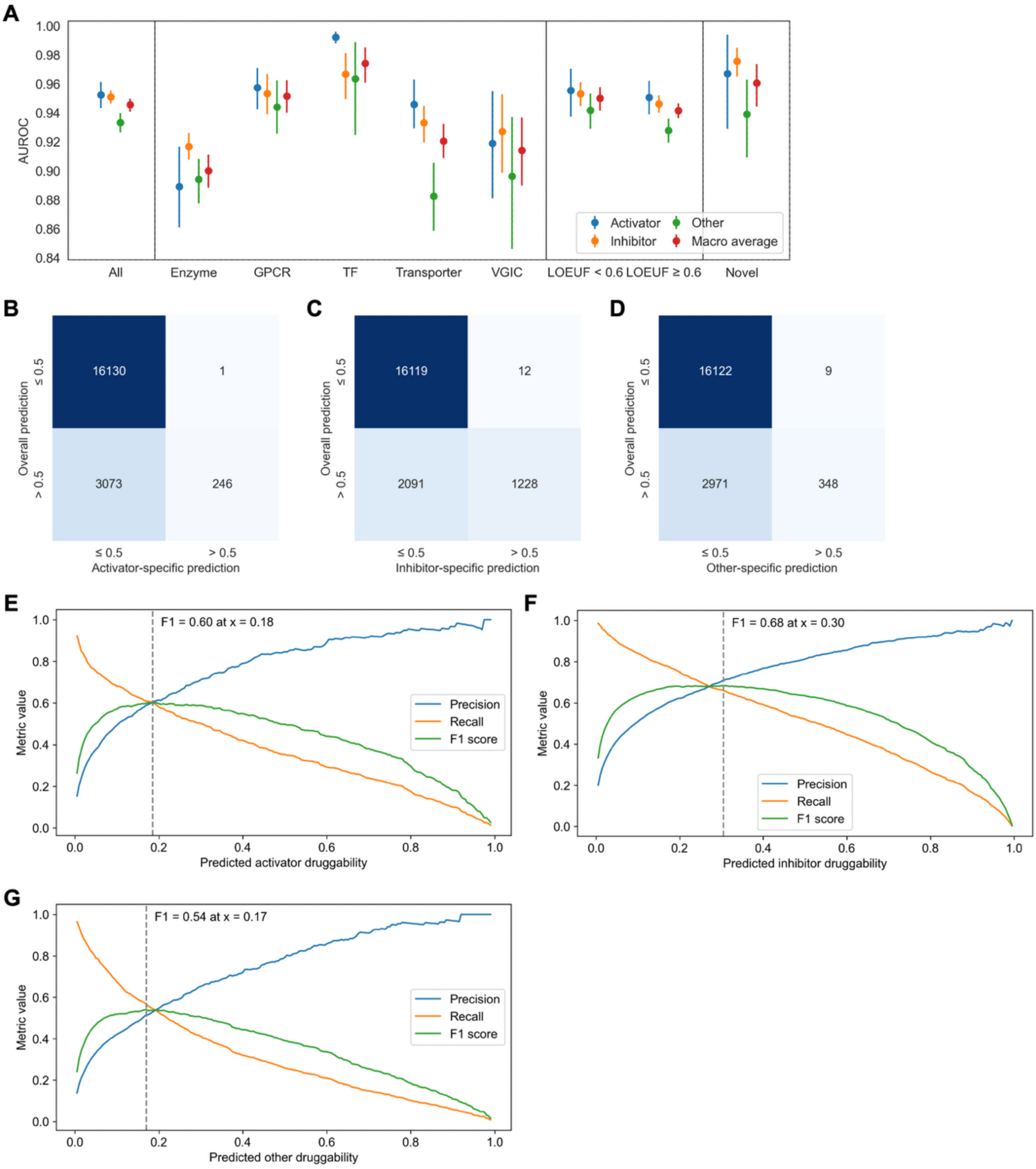

(A) Areas under the receiver operating characteristic curve (AUROC) for DOE-specific druggability predictions across different gene categories, including all protein-coding genes, enzymes, G protein-coupled receptors (GPCRs), transcription factors (TFs), transporters, voltage-gated ion channels (VGICs), constraint groups (LOEUF < 0.6 versus LOEUF ≥ 0.6), and genes with above-median PHAROS novelty scores (score > 0.038). Error bars represent 95% confidence intervals. **(B-D)** Concordance between overall and DOE-specific druggability predictions. **(E-G)** Precision, recall, and F_1 score as a function of predicted DOE-specific druggability, with dashed lines representing the cutoffs yielding maximum F_1 scores. All plots represent holdout performance among 19,450 protein-coding genes.

Figure S6: Overall druggability predictions also predict clinical trial success

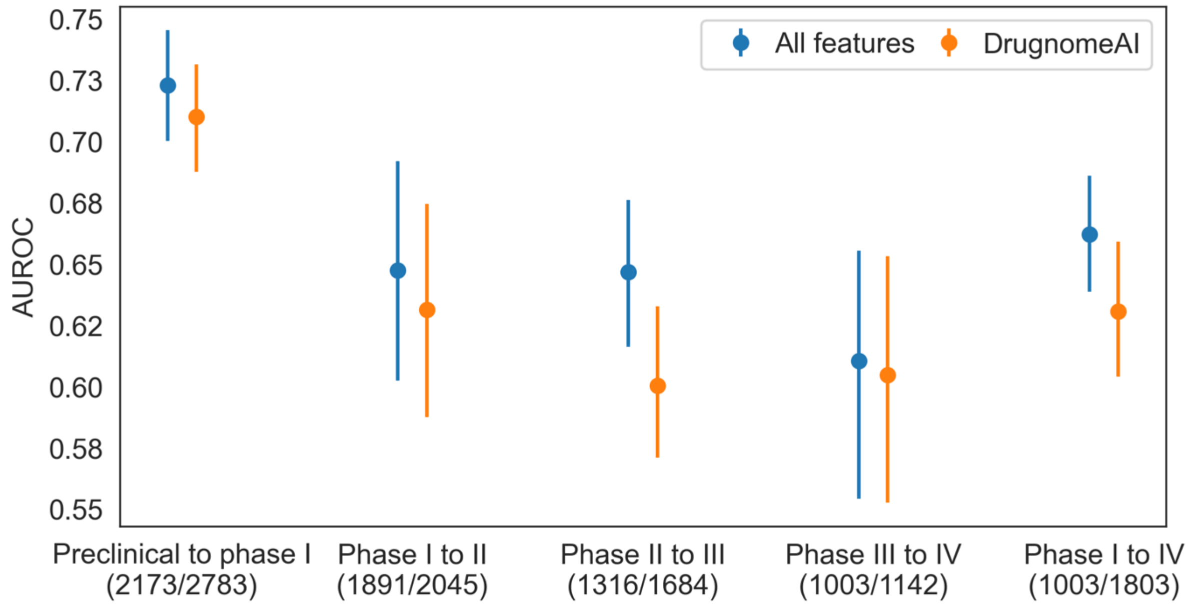

Areas under the receiver operating characteristic curve (AUROC) for predicting the progression of drug targets through clinical trial phases for overall druggability predictions using all features and DrugnomeAI. Numbers in parentheses indicate the number of successes out of the number of targets reaching each clinical trial phase. Error bars represent 95% confidence intervals.

Figure S7: Correlation of predicted druggability with target-disease association scores

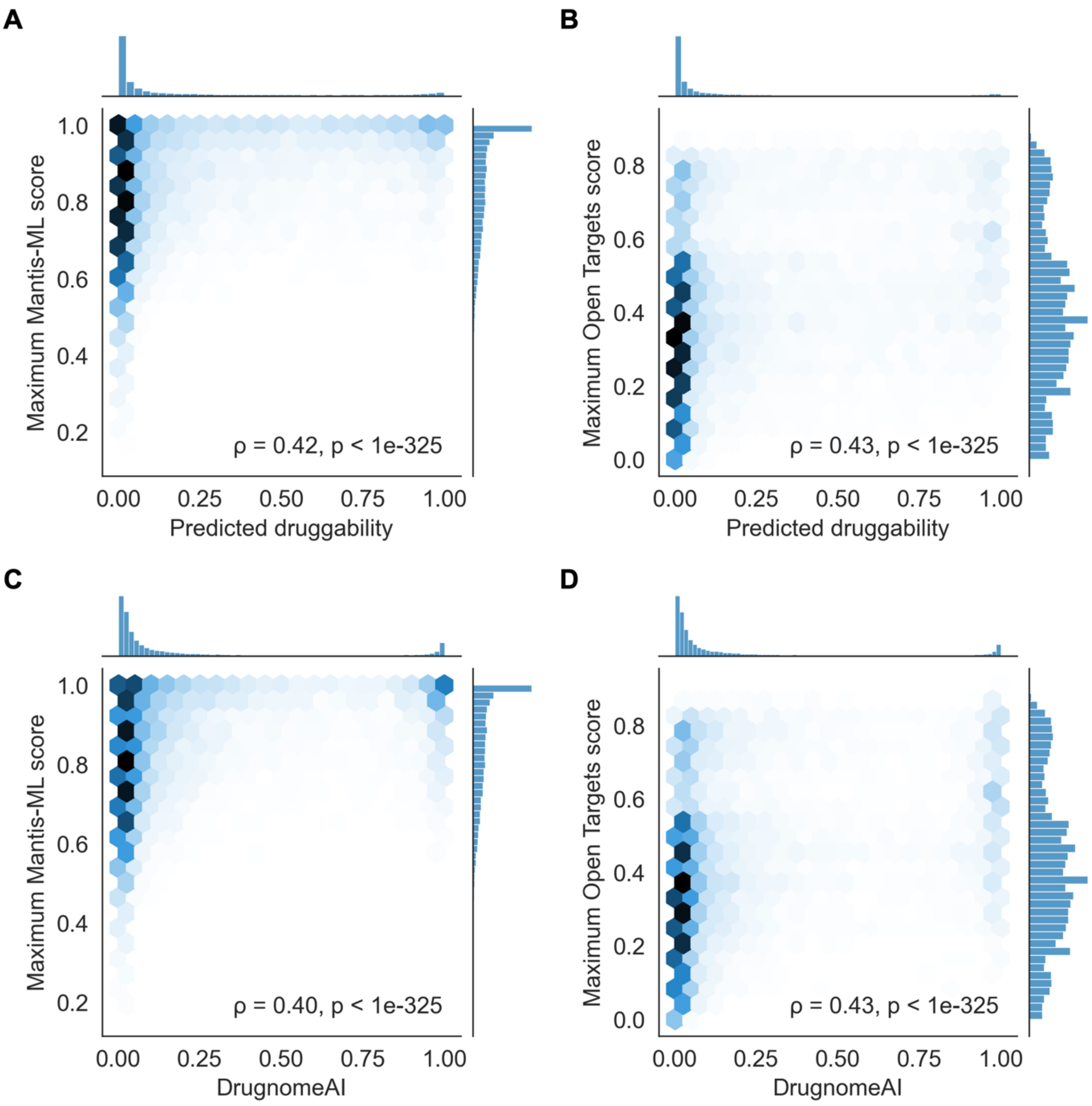

(A-B) Predicted druggability versus maximum per-gene Mantis-ML (**A**; n = 16,994 genes) and Open Targets (**B**; n = 19,450 genes) scores. **(C-D)** DrugnomeAI scores versus maximum per-gene Mantis-ML (**C**; n = 16,878 genes) and Open Targets (**D**; n = 18,379 genes) scores. In **A-D**, predicted druggability or DrugnomeAI scores (x-axis) are positively correlated with Mantis-ML or Open Targets scores representing target-disease associations (y-axis). Hexagonal binning represents density; marginal histograms show score distributions. Text in the lower right of each plot shows Spearman's ρ and p-value.

Figure S8: Performance metrics for DOE predictions

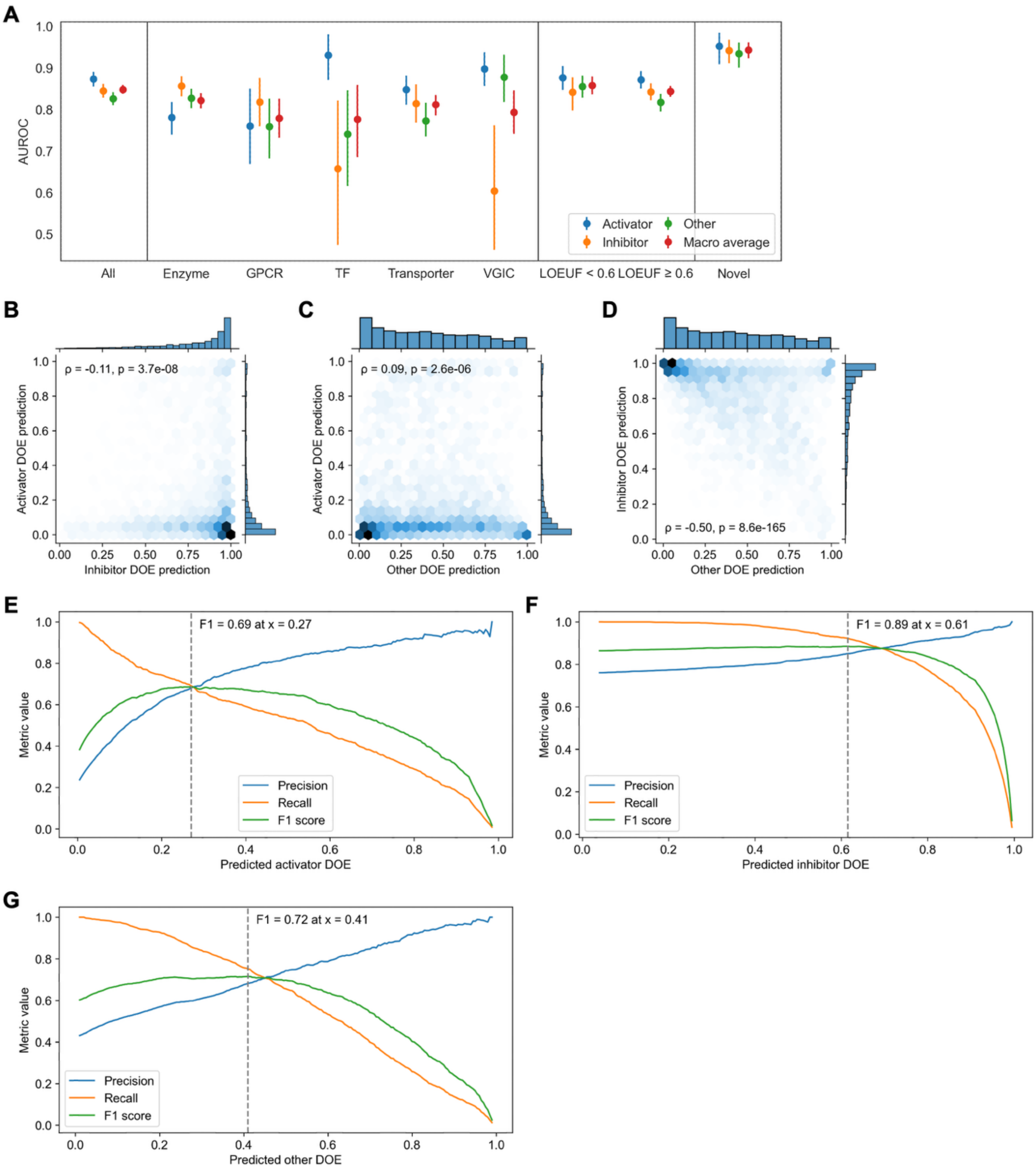

(A) Areas under the receiver operating characteristic curve (AUROC) for DOE predictions across different gene categories, including all protein-coding genes, enzymes, G protein-coupled receptors (GPCRs), transcription factors (TFs), transporters, voltage-gated ion channels (VGICs), constraint groups (LOEUF < 0.6 versus LOEUF ≥ 0.6), and genes with above-median PHAROS novelty scores (score > 0.038). **(B-D)** Correlations between activator, inhibitor, and other mechanism predictions. Hexagonal binning represents density; marginal histograms show score distributions. Text in the lower left of each plot shows Spearman's ρ and p-value. **(E-G)** Precision, recall, and F_1 score as a function of predicted DOE, with dashed lines representing the cutoffs yielding maximum F_1 scores. All plots represent holdout performance among 2,553 known druggable genes.

Figure S9: Gene set enrichment analysis of DOE predictions

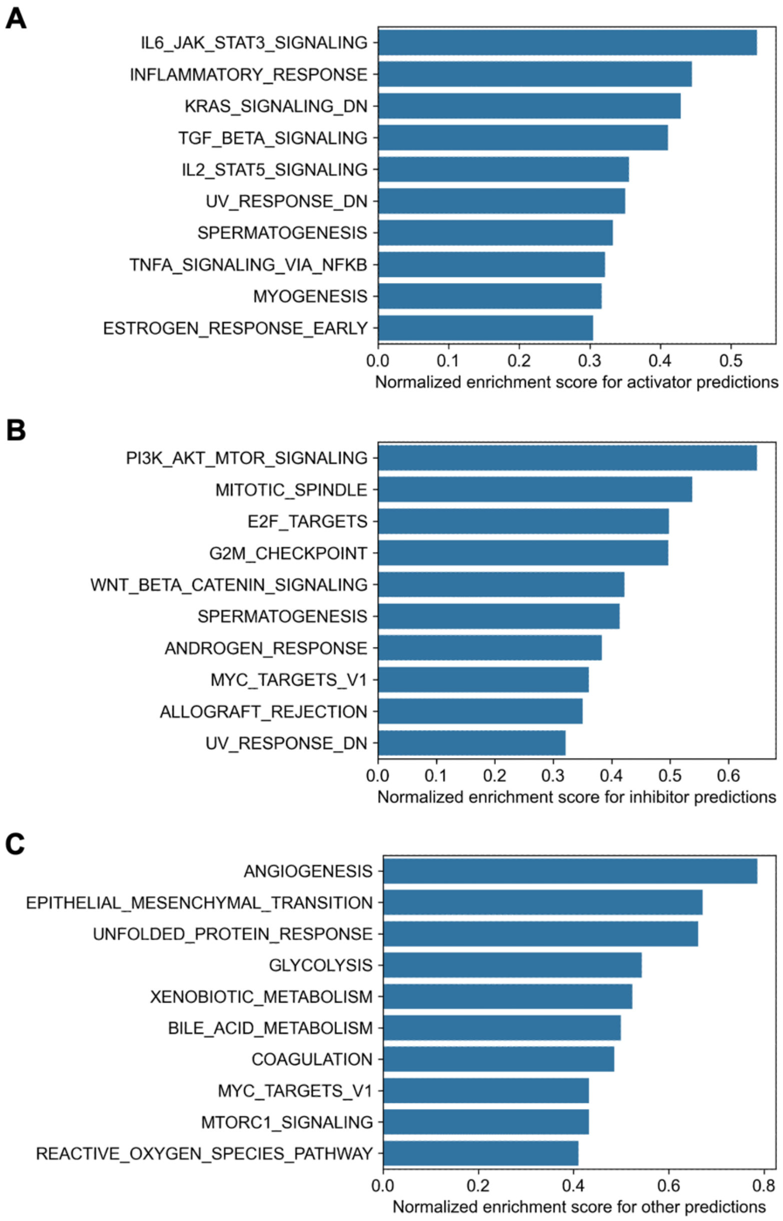

We performed single-sample gene set enrichment analysis on predicted DOE using hallmark gene sets from the Molecular Signatures Database. **(A)** Gene sets enriched with inhibitor predictions. **(B)** Gene sets enriched with activator predictions. **(C)** Gene sets enriched with other mechanism predictions. The normalized enrichment score accounts for differences in gene set size and correlations with the expression dataset, allowing for comparison across gene sets.

Figure S10: Genetic evidence supports DOE predictions across allele frequency bins and data sources

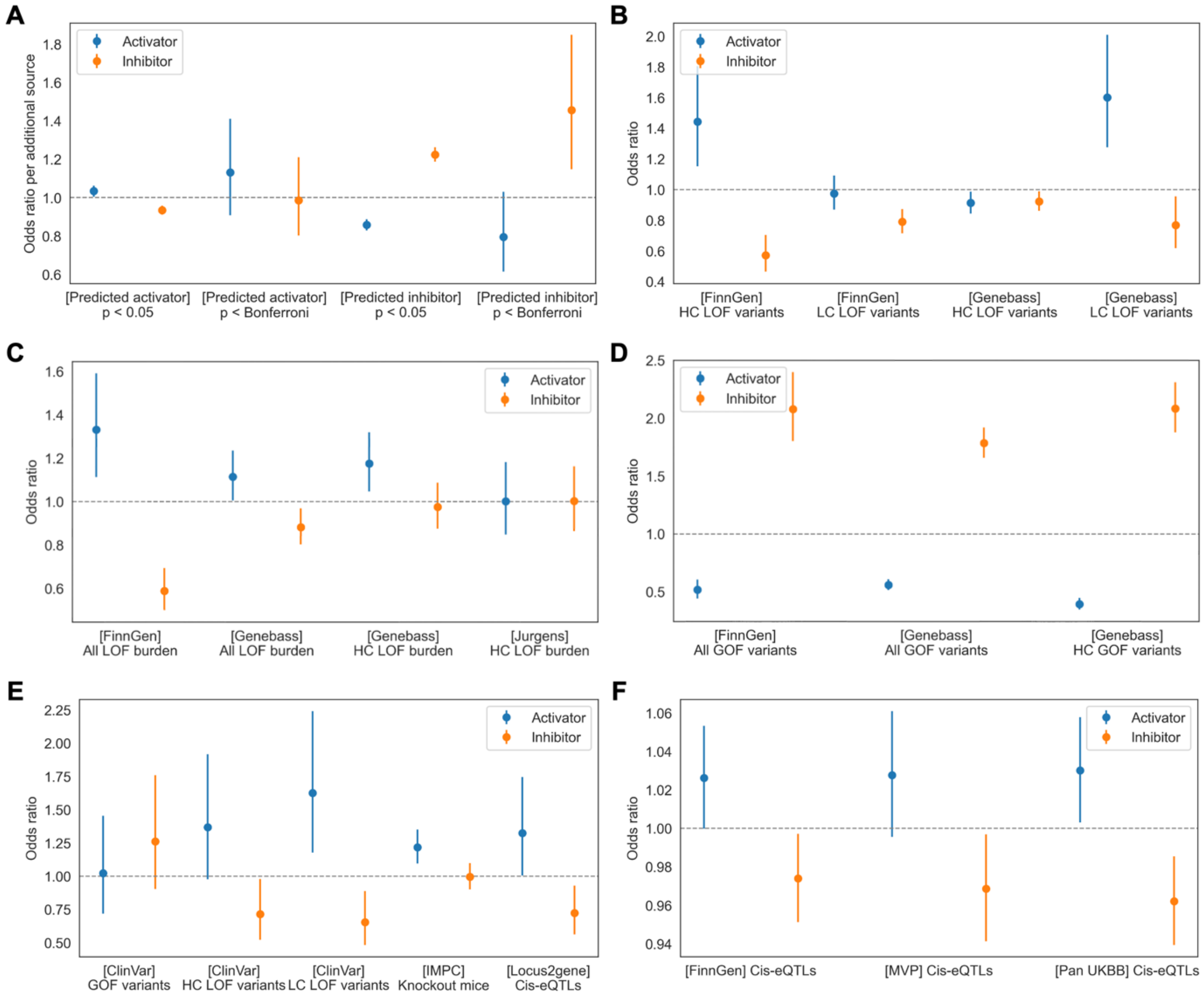

(A) Odds ratios for activator and inhibitor mechanisms per each additional allele frequency bin (common, rare, ultrarare) supporting each mechanism. **(B)** Odds ratios for activator and inhibitor mechanisms stratified by loss-of-function (LOF) variant sources. **(C)** Odds ratios for activator and inhibitor mechanisms stratified by gene burden testing sources. **(D)** Odds ratios for activator and inhibitor mechanisms stratified by gain-of-function (GOF) variant sources. **(E)** Odds ratios for activator and inhibitor mechanisms stratified by Open Targets sources, including clinical variants (ClinVar), mouse knockout phenotypes (IMPC), and Locus2gene. **(F)** Odds ratios for activator and inhibitor mechanisms stratified by common variant sources integrated with expression quantitative trait loci (eQTL) data. For **B-D**, we encoded independent variables as follows: +1 if there was a variant with $p < 0.05$ and a positive beta; -1 if there was a variant with $p < 0.05$ and a negative beta; and 0 otherwise. For **B-E**, high-confidence (HC) and low-confidence (LC) LOF annotations are from LOFTEE. For **C**, we encoded independent variables as follows: +1 if there was a gene burden test with $p < 0.05$ and a positive beta; -1 if there was a gene burden test with $p < 0.05$ and a negative beta; and 0 otherwise. For **F**, we encoded independent variables as follows: +1 if there was a variant with $p < 0.05$ and a beta in the opposite direction as the eQTL beta (predicting an activator mechanism); -1 if there was a variant with $p < 0.05$ and a beta in the same direction

as the eQTL beta (predicting an inhibitor mechanism); and 0 otherwise. Error bars represent 95% confidence intervals.

Figure S11: Performance metrics for gene-disease-specific DOE predictions

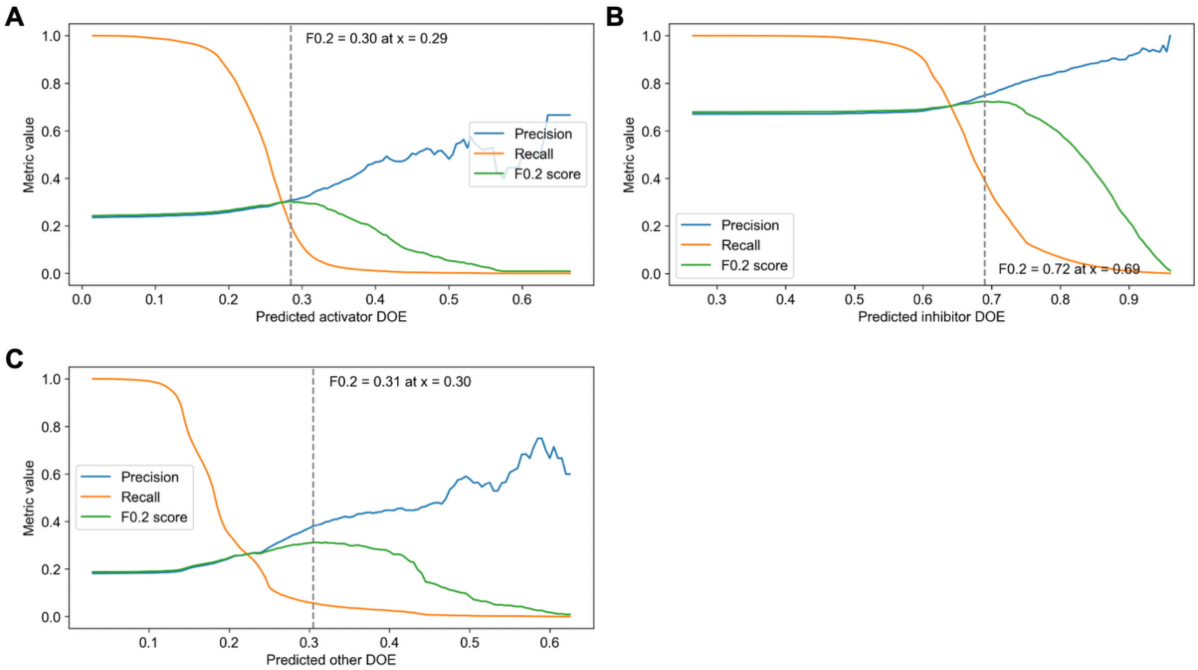

Precision, recall, and $F_{0.2}$ score as a function of predicted DOE, with dashed lines representing the cutoffs yielding maximum $F_{0.2}$ scores. We used $F_{0.2}$ scores to place greater weight on precision compared to recall due to modest model performance. All plots represent holdout performance among 47,822 gene-disease pairs.

Figure S12: Correlation between gene-level and gene-disease-specific DOE predictions

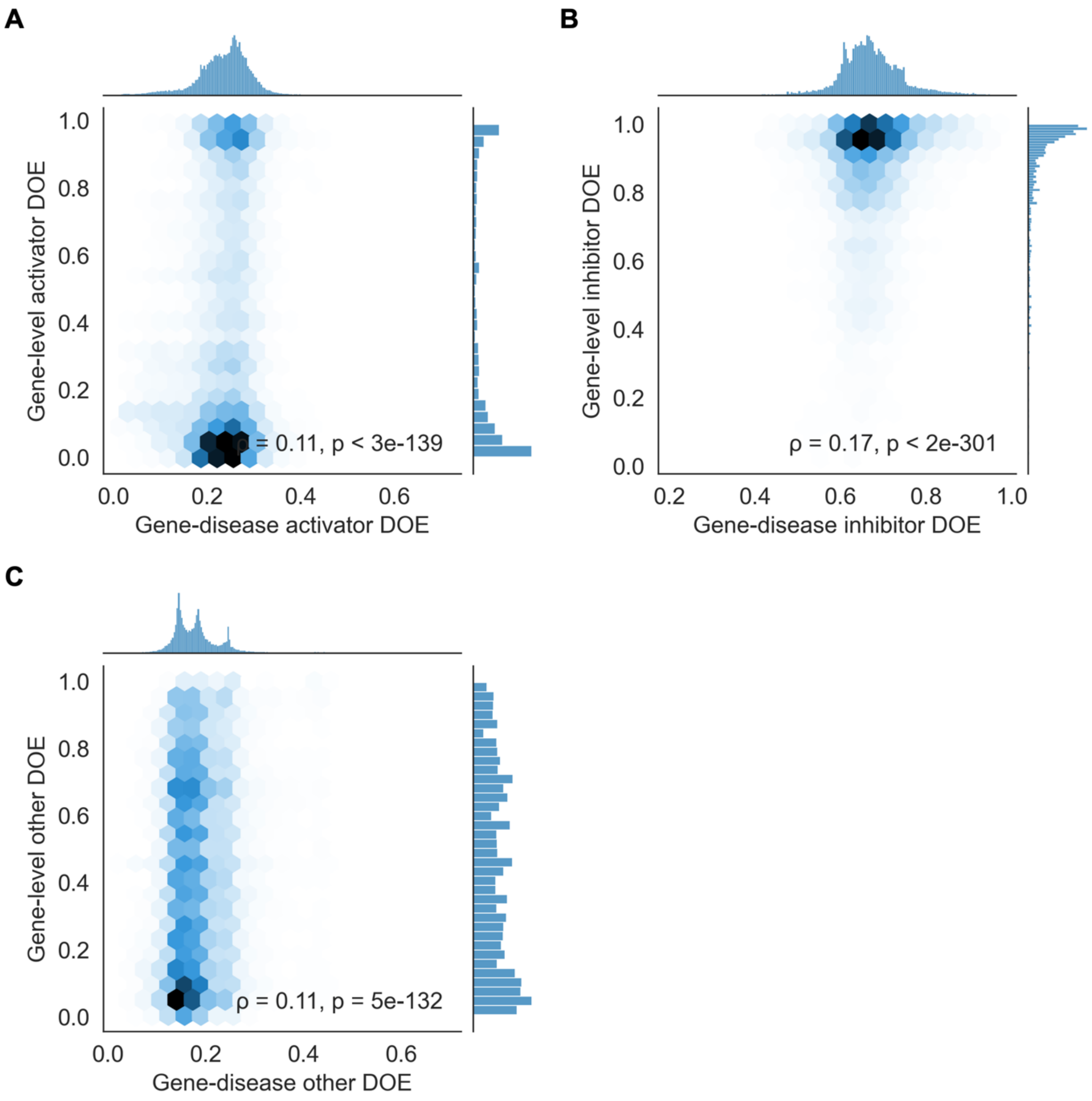

Scatterplots comparing gene-level and gene-disease-specific DOE predictions for activator **(A)**, inhibitor **(B)**, and other **(C)** drug mechanisms among 47,822 gene-disease pairs with indicated drugs. For gene-level DOE, all gene-disease pairs representing the same gene have the same prediction. Hexagonal binning represents density; marginal histograms show score distributions. Text in the lower right of each plot shows Spearman's ρ and p-value.

Figure S13: Comparison of gene-level and gene-disease-specific DOE models

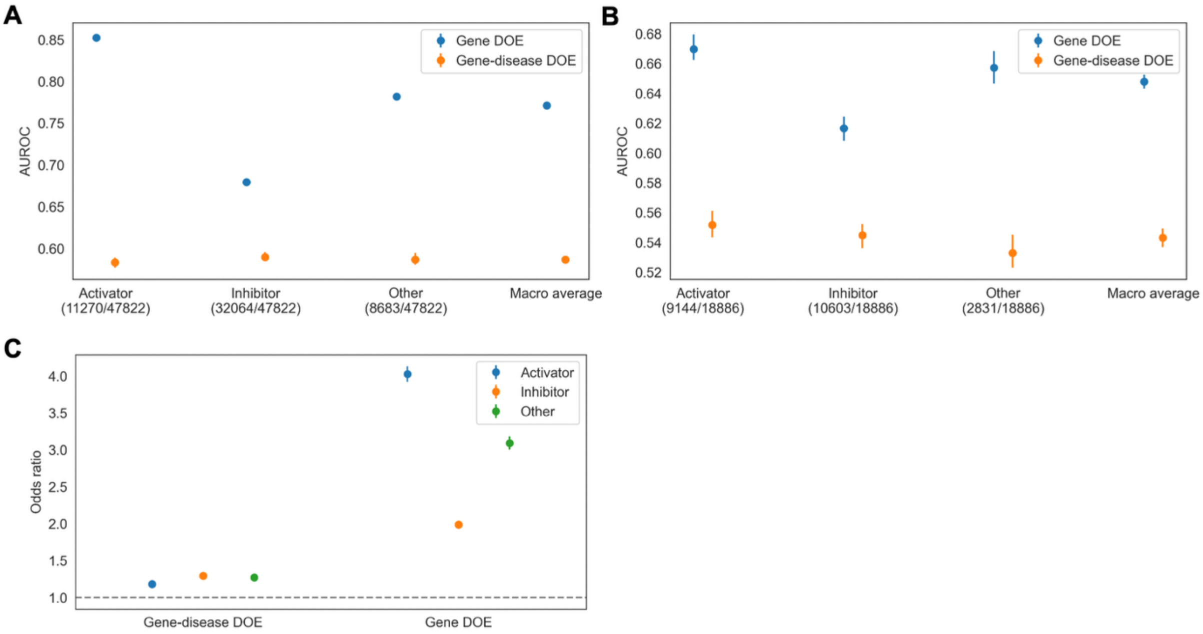

(A, B) Areas under the receiver operating characteristic curve (AUROC) for predicting gene-disease DOE using gene-level and gene-disease DOE models across all gene-disease pairs **(A)** and among genes targeted by both activator and inhibitor drugs **(B)**. **(C)** Odds ratios for gene-level and gene-disease DOE predictions in multivariate logistic regression models. We performed separate regressions for each DOE outcome (activator, inhibitor, other).

Figure S14: Optimization of gene and protein embeddings

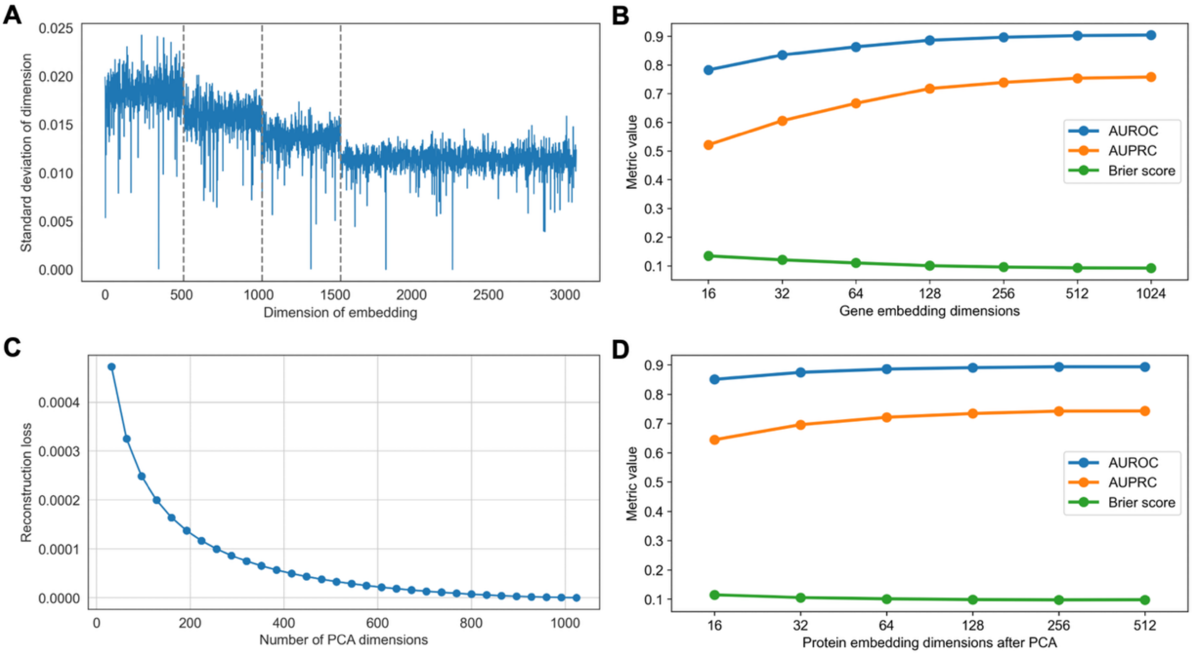

(A) Information density across GenePT embedding dimensions. Dashed lines represent 512, 1,024, and 1,536 dimensions. **(B)** Model performance for predicting overall druggability as a function of gene embedding dimensionality, measured by area under the receiver operating characteristic curve (AUROC), area under the precision-recall curve (AUPRC), and Brier score. **(C)** Reconstruction loss (measured as mean squared error between the original embeddings and the reduced dimensions) as a function of the number of principal component analysis (PCA) components for protein embeddings. **(D)** Model performance for predicting overall druggability as a function of protein embedding dimensionality after PCA.

Figure S15: UMAP visualization of gene and protein embeddings

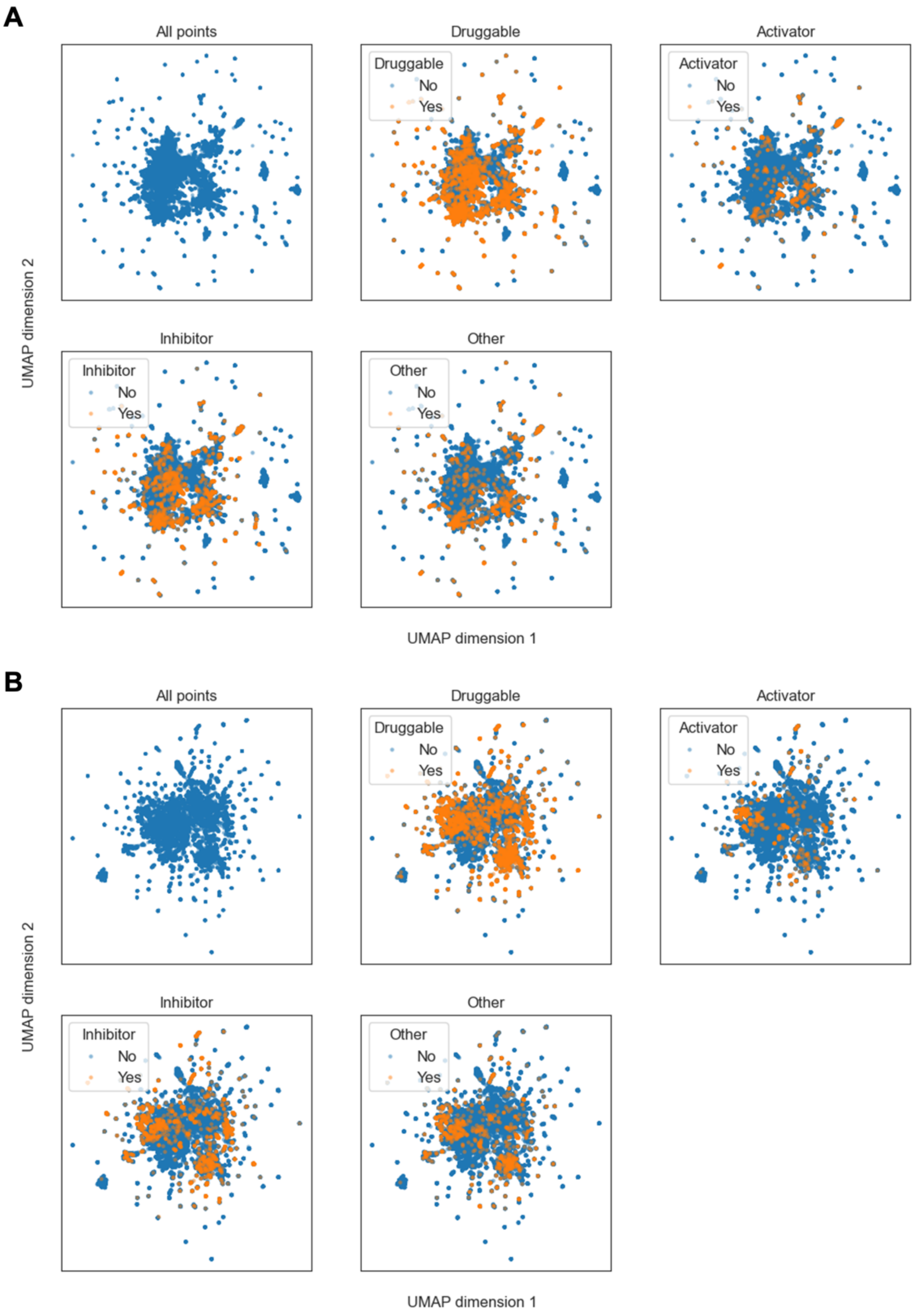

We used Uniform Manifold Approximation and Projection (UMAP) to generate two-dimensional representations of gene and protein embeddings. **(A)** Gene embeddings colored by druggability and DOE. **(B)** Protein embeddings colored by druggability and DOE.

Table S1: Metrics for predicting overall druggability

Outcome	Model	AUROC (95% CI)	AUPRC (95% CI)	Brier score (95% CI)	Proportion positive
All druggable	Tabular features	0.9 (0.895-0.904)	0.74 (0.727-0.753)	0.096 (0.093-0.098)	4096/19450 (21.1%)
	Gene embeddings	0.896 (0.891-0.901)	0.739 (0.725-0.752)	0.095 (0.093-0.098)	
	Protein embeddings	0.89 (0.884-0.895)	0.734 (0.721-0.747)	0.098 (0.095-0.101)	
	All embeddings	0.922 (0.918-0.927)	0.798 (0.786-0.808)	0.085 (0.082-0.087)	
	All features	0.937 (0.933-0.94)	0.83 (0.819-0.84)	0.076 (0.074-0.079)	
	DrugnomeAI	0.905 (0.9-0.911)	0.806 (0.795-0.817)	0.088 (0.085-0.091)	
Tclin + Tchem	Tabular features	0.912 (0.906-0.918)	0.677 (0.658-0.696)	0.068 (0.066-0.071)	2563/19450 (13.2%)
	Gene embeddings	0.907 (0.901-0.914)	0.676 (0.659-0.694)	0.069 (0.066-0.071)	
	Protein embeddings	0.897 (0.89-0.904)	0.688 (0.671-0.706)	0.068 (0.065-0.071)	
	All embeddings	0.933 (0.928-0.938)	0.759 (0.744-0.774)	0.06 (0.057-0.062)	
	All features	0.949 (0.945-0.953)	0.801 (0.787-0.814)	0.053 (0.051-0.056)	
	DrugnomeAI	0.944 (0.939-0.949)	0.833 (0.82-0.846)	0.057 (0.055-0.06)	

Abbreviations: AUROC, area under the receiver operating characteristic curve; AUPRC, area under the precision-recall curve; CI, confidence interval.

Table S2: Metrics for predicting DOE-specific druggability

Outcome	Model	AUROC (95% CI)	AUPRC (95% CI)	Brier score (95% CI)	Proportion positive
Activator druggability	Tabular features	0.92 (0.91-0.931)	0.393 (0.352-0.438)	0.023 (0.021-0.024)	592/19450 (3.0%)
	Gene embeddings	0.928 (0.915-0.94)	0.556 (0.515-0.596)	0.019 (0.018-0.021)	
	Protein embeddings	0.898 (0.882-0.913)	0.523 (0.481-0.565)	0.02 (0.018-0.022)	
	All embeddings	0.939 (0.929-0.949)	0.613 (0.573-0.652)	0.018 (0.017-0.02)	
	All features	0.952 (0.943-0.961)	0.638 (0.599-0.676)	0.017 (0.016-0.019)	
	DrugnomeAI	0.933 (0.925-0.941)	0.316 (0.284-0.349)	0.107 (0.104-0.111)	
Inhibitor druggability	Tabular features	0.913 (0.906-0.919)	0.607 (0.585-0.629)	0.058 (0.055-0.06)	1937/19450 (10.0%)
	Gene embeddings	0.916 (0.909-0.923)	0.652 (0.628-0.673)	0.054 (0.052-0.057)	
	Protein embeddings	0.896 (0.888-0.904)	0.629 (0.606-0.651)	0.057 (0.054-0.059)	
	All embeddings	0.937 (0.931-0.942)	0.726 (0.705-0.744)	0.049 (0.047-0.051)	
	All features	0.951 (0.946-0.955)	0.755 (0.735-0.773)	0.045 (0.043-0.047)	
	DrugnomeAI	0.929 (0.923-0.935)	0.687 (0.664-0.71)	0.072 (0.069-0.075)	
Other druggability	Tabular features	0.883 (0.873-0.892)	0.402 (0.37-0.432)	0.042 (0.04-0.044)	1094/19450 (5.6%)
	Gene embeddings	0.882 (0.872-0.893)	0.441 (0.408-0.47)	0.041 (0.038-0.043)	
	Protein embeddings	0.884 (0.873-0.894)	0.476 (0.445-0.508)	0.039 (0.037-0.041)	
	All embeddings	0.913 (0.905-0.921)	0.543 (0.511-0.572)	0.037 (0.035-0.039)	
	All features	0.933 (0.927-0.94)	0.578 (0.549-0.607)	0.035 (0.033-0.038)	
	DrugnomeAI	0.824 (0.811-0.836)	0.236 (0.215-0.258)	0.119 (0.115-0.123)	
Macro-average	Tabular features	0.906 (0.899-0.911)	0.467 (0.445-0.49)	0.041 (0.039-0.042)	N/A
	Gene embeddings	0.909 (0.902-0.916)	0.55 (0.527-0.571)	0.038 (0.037-0.04)	
	Protein embeddings	0.893 (0.885-0.9)	0.543 (0.519-0.565)	0.039 (0.037-0.04)	
	All embeddings	0.93 (0.924-0.935)	0.627 (0.607-0.647)	0.035 (0.033-0.036)	
	All features	0.945 (0.941-0.95)	0.657 (0.637-0.676)	0.033 (0.031-0.034)	
	DrugnomeAI	0.895 (0.889-0.902)	0.413 (0.396-0.432)	0.099 (0.096-0.103)	

Micro-average	Tabular features	0.913 (0.908-0.918)	0.516 (0.498-0.536)	0.041 (0.039-0.042)	N/A
	Gene embeddings	0.917 (0.911-0.922)	0.577 (0.557-0.597)	0.038 (0.037-0.04)	
	Protein embeddings	0.903 (0.897-0.909)	0.568 (0.547-0.588)	0.039 (0.037-0.04)	
	All embeddings	0.936 (0.931-0.94)	0.656 (0.637-0.673)	0.035 (0.033-0.036)	
	All features	0.95 (0.946-0.954)	0.687 (0.669-0.702)	0.033 (0.031-0.034)	
	DrugnomeAI	0.891 (0.885-0.898)	0.398 (0.382-0.417)	0.099 (0.096-0.103)	

Abbreviations: AUROC, area under the receiver operating characteristic curve; AUPRC, area under the precision-recall curve; CI, confidence interval.

Table S3: Metrics for predicting DOE

Outcome	Model	AUROC (95% CI)	AUPRC (95% CI)	Brier score (95% CI)	Proportion positive
Activator	Tabular features	0.796 (0.774-0.817)	0.592 (0.547-0.64)	0.134 (0.125-0.144)	592/2553 (23.2%)
	Gene embeddings	0.855 (0.833-0.873)	0.717 (0.68-0.752)	0.112 (0.103-0.121)	
	Protein embeddings	0.832 (0.811-0.853)	0.713 (0.677-0.748)	0.114 (0.105-0.123)	
	All embeddings	0.868 (0.849-0.886)	0.754 (0.721-0.787)	0.104 (0.095-0.113)	
	All features	0.873 (0.855-0.891)	0.75 (0.715-0.784)	0.104 (0.096-0.112)	
	DrugnomeAI	0.69 (0.667-0.712)	0.376 (0.343-0.413)	0.454 (0.436-0.469)	
Inhibitor	Tabular features	0.759 (0.737-0.78)	0.903 (0.89-0.917)	0.154 (0.145-0.163)	1937/2553 (75.9%)
	Gene embeddings	0.83 (0.814-0.848)	0.936 (0.925-0.945)	0.136 (0.128-0.145)	
	Protein embeddings	0.799 (0.78-0.819)	0.921 (0.908-0.933)	0.144 (0.135-0.152)	
	All embeddings	0.847 (0.829-0.864)	0.943 (0.934-0.952)	0.131 (0.123-0.14)	
	All features	0.845 (0.828-0.863)	0.941 (0.931-0.951)	0.131 (0.122-0.14)	
	DrugnomeAI	0.749 (0.727-0.77)	0.889 (0.872-0.904)	0.197 (0.185-0.209)	
Other	Tabular features	0.726 (0.706-0.747)	0.681 (0.654-0.712)	0.206 (0.199-0.213)	1094/2553 (42.9%)
	Gene embeddings	0.8 (0.784-0.816)	0.763 (0.738-0.788)	0.179 (0.172-0.186)	
	Protein embeddings	0.788 (0.771-0.805)	0.748 (0.723-0.772)	0.184 (0.176-0.192)	
	All embeddings	0.818 (0.803-0.834)	0.784 (0.76-0.808)	0.171 (0.163-0.178)	
	All features	0.826 (0.812-0.842)	0.791 (0.768-0.813)	0.167 (0.159-0.175)	
	DrugnomeAI	0.382 (0.36-0.403)	0.371 (0.349-0.397)	0.535 (0.518-0.55)	
Macro-average	Tabular features	0.76 (0.748-0.774)	0.725 (0.708-0.745)	0.165 (0.16-0.17)	N/A
	Gene embeddings	0.828 (0.817-0.84)	0.805 (0.79-0.821)	0.142 (0.137-0.148)	
	Protein embeddings	0.806 (0.793-0.819)	0.794 (0.779-0.809)	0.147 (0.141-0.153)	
	All embeddings	0.844 (0.833-0.855)	0.827 (0.813-0.841)	0.135 (0.13-0.141)	
	All features	0.848 (0.837-0.86)	0.827 (0.813-0.842)	0.134 (0.128-0.139)	
	DrugnomeAI	0.607 (0.597-0.618)	0.545 (0.531-0.562)	0.395 (0.387-0.402)	
Micro-average	Tabular features	0.836 (0.827-0.846)	0.823 (0.811-0.836)	0.165 (0.16-0.17)	N/A
	Gene embeddings	0.877 (0.868-0.885)	0.869 (0.859-0.879)	0.142 (0.137-0.148)	
	Protein embeddings	0.868 (0.859-0.877)	0.858 (0.845-0.869)	0.147 (0.141-0.153)	
	All embeddings	0.888 (0.879-0.896)	0.882 (0.873-0.892)	0.135 (0.13-0.141)	
	All features	0.89 (0.882-0.899)	0.882 (0.872-0.892)	0.134 (0.128-0.139)	

DrugnomeAI	0.567 (0.559-0.577)	0.531 (0.517-0.548)	0.395 (0.387-0.402)

Abbreviations: AUROC, area under the receiver operating characteristic curve; AUPRC, area under the precision-recall curve; CI, confidence interval.

Table S4: Correlations between different predictions

Outcome	Model	Spearman's ρ	p-value
Activator	DOE-specific druggability	0.50	2.66E-164
	DOE only	0.19	3.18E-21
Inhibitor	DOE-specific druggability	0.81	0.00E+00
	DOE only	0.44	1.77E-122
Other	DOE-specific druggability	0.50	1.66E-160
	DOE only	-0.08	1.99E-05

We calculated Spearman correlations of overall druggability predictions with either (1) DOE-specific druggability predictions or (2) isolated DOE predictions among 2,553 druggable genes. We did this separately for activator, inhibitor, and other mechanism predictions.

Table S5: Single sample gene set enrichment with DOE predictions

Gene set	Activator	Inhibitor	Other
ADIPOGENESIS	0.03	-0.30	0.34
ALLOGRAFT_REJECTION	0.26	0.35	0.32
ANDROGEN_RESPONSE	-0.03	0.38	0.04
ANGIOGENESIS	0.17	-0.06	0.79
APICAL_JUNCTION	0.17	0.27	0.17
APOPTOSIS	0.30	0.21	0.30
BILE_ACID_METABOLISM	0.21	-0.22	0.50
CHOLESTEROL_HOMEOSTASIS	0.18	-0.20	0.35
COAGULATION	0.20	-0.04	0.49
COMPLEMENT	0.12	0.07	0.38
DNA_REPAIR	-0.29	0.13	-0.04
E2F_TARGETS	-0.28	0.50	-0.02
EPITHELIAL_MESENCHYMAL_TRANSITION	0.05	-0.33	0.67
ESTROGEN_RESPONSE_EARLY	0.30	0.02	0.21
ESTROGEN_RESPONSE_LATE	0.14	0.13	0.14
FATTY_ACID_METABOLISM	-0.05	-0.35	0.38
G2M_CHECKPOINT	-0.18	0.50	-0.09
GLYCOLYSIS	-0.04	-0.27	0.54
HEME_METABOLISM	0.06	-0.14	0.38
HYPOXIA	0.18	-0.13	0.40
IL2_STAT5_SIGNALING	0.36	-0.14	0.23
IL6_JAK_STAT3_SIGNALING	0.54	0.04	0.21
INFLAMMATORY_RESPONSE	0.44	0.02	0.04
INTERFERON_ALPHA_RESPONSE	0.01	0.22	0.13
INTERFERON_GAMMA_RESPONSE	0.17	0.16	0.18
KRAS_SIGNALING_DN	0.43	0.23	0.35
KRAS_SIGNALING_UP	0.29	0.16	0.22
MITOTIC_SPINDLE	-0.18	0.54	-0.13
MTORC1_SIGNALING	-0.16	-0.02	0.43

MYC_TARGETS_V1	-0.46	0.36	0.43
MYOGENESIS	0.32	-0.06	0.36
OXIDATIVE_PHOSPHORYLATION	-0.29	0.17	-0.03
P53_PATHWAY	0.19	0.28	0.23
PEROXISOME	0.06	-0.13	0.38
PI3K_AKT_MTOR_SIGNALING	0.10	0.65	-0.21
PROTEIN_SECRETION	-0.09	-0.02	0.36
REACTIVE_OXYGEN_SPECIES_PATHWAY	-0.13	-0.05	0.41
SPERMATOGENESIS	0.33	0.41	0.08
TGF_BETA_SIGNALING	0.41	0.20	0.14
TNFA_SIGNALING_VIA_NFKB	0.32	0.21	0.18
UNFOLDED_PROTEIN_RESPONSE	-0.11	-0.18	0.66
UV_RESPONSE_DN	0.35	0.32	0.24
UV_RESPONSE_UP	0.15	0.15	0.14
WNT_BETA_CATENIN_SIGNALING	0.15	0.42	-0.09
XENOBIOTIC_METABOLISM	0.09	-0.27	0.52

Values are normalized enrichment scores.

Table S6: Disease relevance of gene-level DOE predictions

DOE	Percentile cutoff	Raw score	Supported/total above cutoff	Supported/total below cutoff	Odds ratio (95% CI)	p-value
Activator	25	0.03	595/1183	2387/3549	2.03 (1.78-2.32)	3.35E-25
	50	0.06	1302/2366	1680/2366	2 (1.77-2.26)	1.13E-29
	75	0.18	2117/3549	865/1183	1.84 (1.59-2.13)	1.67E-16
Inhibitor	25	0.67	177/1183	687/3549	1.36 (1.14-1.63)	7.26E-04
	50	0.85	392/2366	472/2366	1.25 (1.08-1.46)	2.64E-03
	75	0.94	617/3549	247/1183	1.25 (1.06-1.48)	7.14E-03

This table analyzes 2,553 known druggable genes and 2,179 additional predicted druggable genes (4,732 total genes). We retrieved DOE assessments from Open Targets across seven sources: Locus2Gene, gene burden, IMPC, Orphanet, Gene2Phenotype, ClinVar germline, and ClinVar somatic. For each gene-disease-DOE triplet, we first computed a harmonic sum within each source (e.g., aggregating variant-level evidence in ClinVar), then computed a second harmonic sum across sources to obtain a single score. We collapsed these scores by gene-DOE pair and defined a pair as having an Open Targets assessment if the score was ≥ 0.3. To test for concordance, we used logistic regression to evaluate whether gene-level DOE predictions were associated with having an Open Targets DOE assessment for any disease. Abbreviations: CI, confidence interval.

Table S7: Correlations of gene-disease DOE predictions with association scores

Target-disease association score	DOE score	Spearman's ρ	p-value
Mantis-ML	Activator	-0.03	1.88E-13
	Inhibitor	0.00	5.24E-01
	Other	0.00	7.97E-01
Open Targets	Activator	0.04	2.25E-21
	Inhibitor	0.06	4.63E-48
	Other	0.00	2.65E-01

Table S8: Examples of novel targets with confident gene-disease-specific DOE predictions

Gene	Disease	Disease description	Target-disease association scores		Gene-disease-specific DOE predictions			
			Mantis-ML	Open Targets	Activator	Inhibitor	Other	Non-zero genetic features
CFH	H35	Other retinal disorders	0.90	0.67	0.82	0.31	0.36	17
MC4R	E11	Type 2 diabetes mellitus	0.90	0.51	0.74	0.39	0.23	9
TERT	J84	Other interstitial pulmonary diseases	N/A	0.69	0.72	0.28	0.36	16
NLRP3	L53	Other erythematous conditions	0.91	0.01	0.02	0.97	0.04	6
KIT	Q61	Cystic kidney disease	0.97	0.07	0.03	0.95	0.06	6

These selected gene-disease pairs have strong target-disease association evidence (Mantis-ML score > 0.9 or Open Targets score > 0.3), are known or predicted to be druggable, and do not have drugs under clinical investigation or approved. We used a higher cutoff for Mantis-ML as it is a machine learning score, whereas Open Targets is based on empirical genetics and experimental evidence.

Table S9: Genes with simultaneously high (> 0.9) activator and inhibitor gene-level DOE predictions

Gene	Activator	Inhibitor	Other
ADORA1	0.95	0.93	0.66
ADORA2A	0.95	0.99	0.79
ADORA3	0.95	0.93	0.53
ADRA1A	0.97	0.99	0.89
ADRA1B	0.97	0.99	0.70
ADRA1D	0.98	0.99	0.82
ADRA2A	0.97	0.99	0.93
ADRA2B	0.93	0.95	0.66
ADRA2C	0.96	0.97	0.80
ADRB1	0.97	0.99	0.62
ADRB2	0.98	0.99	0.67
AVPR1B	0.95	0.93	0.48
CHRM1	0.96	0.98	0.64
CHRM2	0.98	0.99	0.67
CHRM3	0.97	0.95	0.45
CHRM4	0.98	0.99	0.76
CHRM5	0.97	0.95	0.52
CHRNA1	0.96	0.96	0.25
CHRNA2	0.91	0.96	0.41
CHRNA3	0.93	0.93	0.34
CHRNA4	0.98	0.96	0.11
CHRNA6	0.95	0.95	0.14
CHRNB1	0.99	0.97	0.32
CHRNB2	0.90	0.98	0.22
CHRNB3	0.96	0.97	0.19
CHRNB4	0.93	0.94	0.29
CHRNE	0.95	0.92	0.26
CHRNG	0.96	0.92	0.36
DRD1	0.96	0.99	0.81

DRD2	0.94	0.97	0.90
ESR1	0.96	0.97	0.50
GABRA1	0.99	0.99	0.75
GABRA2	0.98	0.98	0.54
GABRA3	0.98	0.98	0.60
GABRA4	0.92	0.99	0.45
GABRA5	0.98	0.98	0.64
GABRA6	0.90	0.92	0.30
GABRB1	0.97	0.99	0.64
GABRB2	0.99	0.98	0.45
GABRB3	0.95	0.98	0.70
GABRD	0.94	0.99	0.42
GABRG1	0.97	0.95	0.27
GABRG2	0.97	0.98	0.70
GABRG3	0.98	0.95	0.56
GABRP	0.96	0.91	0.17
GABRQ	0.93	0.91	0.47
GABRR1	0.97	0.98	0.36
GABRR2	0.97	0.91	0.55
GABRR3	0.93	0.95	0.77
GLRA1	0.96	0.97	0.64
GLRA2	0.94	0.93	0.61
GLRA3	0.98	0.93	0.48
GLRB	0.98	0.93	0.47
GRIA2	0.96	0.99	0.42
GRM3	0.92	0.96	0.55
GRM4	0.94	0.99	0.32
HRH3	0.96	0.96	0.53
HTR1A	0.94	0.96	0.94
HTR1B	0.96	0.93	0.93
HTR1D	0.98	0.96	0.88
HTR2A	0.99	0.95	0.87

HTR2C	0.96	0.94	0.77
HTR4	0.98	0.95	0.91
HTR5A	0.96	0.93	0.66
HTR7	0.93	0.97	0.81
NR1H2	0.95	0.98	0.48
PPARA	0.96	0.97	0.77
PTGER2	0.92	0.92	0.13
RXRA	0.99	0.95	0.92

Table S10: Odds ratios for gene-disease pairs supported by gene-level and/or gene-disease-specific DOE predictions

DOE	Comparison group	Success/total	Odds ratio (95% CI)	p-value
Activator	Baseline (Gene-disease < 0.29, Gene-level < 0.27)	1237/24838	N/A	
	Gene-disease ≥ 0.29, Gene-level < 0.27	203/2997	1.39 (1.19-1.62)	3.05E-05
	Gene-disease < 0.29, Gene-level ≥ 0.27	8163/16923	17.78 (16.67-18.97)	p < 1E-325
	Gene-disease ≥ 0.29, Gene-level ≥ 0.27	1667/3064	22.77 (20.78-24.94)	p < 1E-325
Inhibitor	Baseline (Gene-disease < 0.69, Gene-level < 0.61)	825/3321	N/A	
	Gene-disease ≥ 0.69, Gene-level < 0.61	359/968	1.78 (1.53-2.08)	9.75E-14
	Gene-disease < 0.69, Gene-level ≥ 0.61	18732/27819	6.24 (5.74-6.77)	p < 1E-325
	Gene-disease ≥ 0.69, Gene-level ≥ 0.61	12148/15714	10.31 (9.45-11.24)	p < 1E-325
Other	Baseline (Gene-disease < 0.3, Gene-level < 0.41)	1383/22755	N/A	
	Gene-disease ≥ 0.3, Gene-level < 0.41	41/505	1.37 (0.99-1.89)	5.95E-02
	Gene-disease < 0.3, Gene-level ≥ 0.41	6786/23685	6.21 (5.84-6.6)	p < 1E-325
	Gene-disease ≥ 0.3, Gene-level ≥ 0.41	473/877	18.09 (15.67-20.88)	p < 1E-325

Odds ratios represent the odds of having the respective DOE in the comparison group relative to the baseline group. In the "Comparison group" column, we used our recommended cutoffs (Table 1) for each predictor. Abbreviations: CI, confidence interval.

Table S11: Classification of drug mechanisms

DOE	Mechanism	Count
Activator	ACTIVATOR	207
	AGONIST	1327
	FULL AGONIST	2
	INDUCER	54
	OPENER	17
	PARTIAL AGONIST	98
	POSITIVE ALLOSTERIC MODULATOR	109
	POSITIVE MODULATOR	35
	POTENTIATOR	22
	STIMULATOR	23
	UPREGULATOR	3
Inhibitor	ALLOSTERIC ANTAGONIST	1
	ANTAGONIST	1348
	ANTISENSE INHIBITOR	35
	ANTISENSE OLIGONUCLEOTIDE	1
	BINDING	1
	BLOCKER	227
	CHANNEL BLOCKER	1
	DEGRADATION	2
	DEGRADER	6
	DOWNREGULATOR	10
	INACTIVATOR	5
	INHIBITION	43
	INHIBITION OF SYNTHESIS	1
	INHIBITOR	3843
	INHIBITORY ALLOSTERIC MODULATOR	2
	INVERSE AGONIST	31
	NEGATIVE ALLOSTERIC MODULATOR	22
	NEGATIVE MODULATOR	18

	NEUTRALIZER	1
	NUCLEOTIDE EXCHANGE BLOCKER	1
	PARTIAL ANTAGONIST	1
	RELEASING AGENT	20
	RNAI INHIBITOR	21
	SUPPRESSOR	3
	TRANSLOCATION INHIBITOR	1
	WEAK INHIBITOR	1
Other	BINDING AGENT	521
	CHELATING AGENT	2
	COFACTOR	31
	CROSS-LINKING AGENT	18
	DISRUPTING AGENT	7
	GENE OR PROTEIN REPLACEMENT	51
	HYDROLYTIC ENZYME	8
	MODULATOR	452
	OTHER SPECIFIED	19
	PROTEOLYTIC ENZYME	3
	STABILISER	27
	SUBSTRATE	55

Table S12: Comparison of model architectures for predicting overall and DOE-specific druggability

Outcome	Architecture	AUROC (95% CI)	AUPRC (95% CI)	Brier score (95% CI)
Overall druggability	CNN	0.926 (0.922-0.93)	0.811 (0.8-0.821)	0.081 (0.078-0.083)
	Logistic regression	0.921 (0.916-0.925)	0.792 (0.78-0.803)	0.085 (0.082-0.088)
	XGBoost	0.937 (0.933-0.94)	0.83 (0.819-0.84)	0.076 (0.074-0.079)
Activator druggability	CNN	0.947 (0.937-0.956)	0.633 (0.594-0.671)	0.017 (0.016-0.019)
	Logistic regression	0.936 (0.926-0.946)	0.559 (0.515-0.602)	0.02 (0.018-0.021)
	XGBoost	0.952 (0.943-0.961)	0.638 (0.599-0.676)	0.017 (0.016-0.019)
Inhibitor druggability	CNN	0.941 (0.936-0.946)	0.725 (0.703-0.745)	0.046 (0.044-0.049)
	Logistic regression	0.932 (0.926-0.937)	0.687 (0.665-0.707)	0.051 (0.048-0.053)
	XGBoost	0.951 (0.946-0.955)	0.755 (0.735-0.773)	0.045 (0.043-0.047)
Other druggability	CNN	0.919 (0.911-0.927)	0.546 (0.516-0.574)	0.036 (0.034-0.038)
	Logistic regression	0.903 (0.894-0.911)	0.481 (0.449-0.511)	0.039 (0.037-0.041)
	XGBoost	0.933 (0.927-0.94)	0.578 (0.549-0.607)	0.035 (0.033-0.038)
Macro-average (activator, inhibitor, other)	CNN	0.936 (0.931-0.941)	0.635 (0.614-0.655)	0.033 (0.032-0.034)
	Logistic regression	0.924 (0.918-0.929)	0.576 (0.552-0.598)	0.036 (0.035-0.038)
	XGBoost	0.945 (0.941-0.95)	0.657 (0.637-0.676)	0.033 (0.031-0.034)
Micro-average (activator, inhibitor, other)	CNN	0.94 (0.936-0.944)	0.66 (0.641-0.677)	0.033 (0.032-0.034)
	Logistic regression	0.93 (0.925-0.934)	0.606 (0.586-0.625)	0.036 (0.035-0.038)
	XGBoost	0.95 (0.946-0.954)	0.687 (0.669-0.702)	0.033 (0.031-0.034)

Abbreviations: AUROC, area under the receiver operating characteristic curve; AUPRC, area under the precision-recall curve; CI, confidence interval.

Table S13: Comparison of DrugnomeAI models for predicting overall druggability

Outcome	Model	AUROC (95% CI)	AUPRC (95% CI)	Brier score (95% CI)
All druggable	Tchem	0.89 (0.884-0.896)	0.765 (0.753-0.777)	0.096 (0.093-0.099)
	Tclin	0.884 (0.877-0.89)	0.763 (0.75-0.774)	0.134 (0.13-0.139)
	Tclin & Tchem	0.905 (0.9-0.911)	0.806 (0.795-0.817)	0.088 (0.085-0.091)
	Tclin & Tier 1	0.891 (0.885-0.897)	0.769 (0.756-0.781)	0.101 (0.097-0.104)
	Tier 1	0.884 (0.878-0.89)	0.755 (0.743-0.768)	0.104 (0.1-0.107)
	Tier 1 & 2	0.903 (0.897-0.908)	0.789 (0.777-0.799)	0.092 (0.089-0.095)
	Tier 1 & 2 & 3A	0.875 (0.868-0.881)	0.74 (0.727-0.754)	0.106 (0.103-0.11)
Tclin + Tchem	Tchem	0.931 (0.926-0.936)	0.767 (0.751-0.783)	0.068 (0.066-0.071)
	Tclin	0.907 (0.9-0.914)	0.736 (0.719-0.752)	0.072 (0.069-0.076)
	Tclin & Tchem	0.944 (0.939-0.949)	0.833 (0.82-0.846)	0.057 (0.055-0.06)
	Tclin & Tier 1	0.914 (0.907-0.92)	0.722 (0.705-0.739)	0.069 (0.067-0.072)
	Tier 1	0.908 (0.901-0.914)	0.696 (0.678-0.713)	0.073 (0.07-0.076)
	Tier 1 & 2	0.925 (0.919-0.93)	0.756 (0.74-0.771)	0.073 (0.07-0.075)
	Tier 1 & 2 & 3A	0.898 (0.891-0.905)	0.68 (0.661-0.697)	0.097 (0.094-0.1)

Abbreviations: AUROC, area under the receiver operating characteristic curve; AUPRC, area under the precision-recall curve; CI, confidence interval.
